# Quantitative Proteomic Analyses Identify ABA-Related Proteins and Signal Pathways in Maize Leaves under Drought Conditions

**DOI:** 10.3389/fpls.2016.01827

**Published:** 2016-12-08

**Authors:** Yulong Zhao, Yankai Wang, Hao Yang, Wei Wang, Jianyu Wu, Xiuli Hu

**Affiliations:** State Key Laboratory of Wheat and Maize Crop Science, Collaborative Innovation Center of Henan Grain Crops, Henan Agricultural UniversityZhengzhou, China

**Keywords:** *Zea mays* L., drought stress, abscisic acid (ABA), quantitative proteome, iTRAQ, LC-MS/MS, ABA signaling pathways

## Abstract

Drought stress is one of major factors resulting in maize yield loss. The roles of abscisic acid (ABA) have been widely studied in crops in response to drought stress. However, more attention is needed to identify key ABA-related proteins and also gain deeper molecular insights about drought stress in maize. Based on this need, the physiology and proteomics of the ABA-deficient maize mutant *vp5* and its wild-type *Vp5* under drought stress were examined and analyzed. Malondialdehyde content increased and quantum efficiency of photosystem II decreased under drought stress in both genotypes. However, the magnitude of the increase or decrease was significantly higher in *vp5* than in *Vp5*. A total of 7051 proteins with overlapping expression patterns among three replicates in the two genotypes were identified by Multiplex run iTRAQ-based quantitative proteomic and liquid chromatography-tandem mass spectrometry methods, of which the expression of only 150 proteins (130 in *Vp5*, 27 in *vp5*) showed changes of at least 1.5-fold under drought stress. Among the 150 proteins, 67 and 60 proteins were up-regulated and down-regulated by drought stress in an ABA-dependent way, respectively. ABA was found to play active roles in regulating signaling pathways related to photosynthesis, oxidative phosphorylation (mainly related to ATP synthesis), and glutathione metabolism (involved in antioxidative reaction) in the maize response to drought stress. Our results provide an extensive dataset of ABA-dependent, drought-regulated proteins in maize plants, which may help to elucidate the underlying mechanisms of ABA-enhanced tolerance to drought stress in maize.

## Introduction

Humans are heavily dependent on cereals as a main food crop. Among cereal crops, maize is widely cultivated all over the world, in addition to rice and wheat. Yet maize production is prominently affected by drought stress, which has been proved to be one of the major stress factors causing yield loss (Gong et al., 2014). It is predicted that the reduction of maize yield will reach 39.3% when water reduction is ~40% (Daryanto et al., 2016). Many drought-tolerant maize varieties have been cultivated by breeding efforts and technology improvement, which can increase the production of crops. Nevertheless, the yield increase may be counteracted by the prolonged drought stress caused by global climate change and uncertainties of precipitation patterns. Food security is therefore becoming more vulnerable in comparison with the past (FAO, 2008). For food security, it is necessary to determine more deeply the effects of drought stress on maize development and physiology, and the molecular mechanisms at different stages.

Both abscisic acid (ABA)-dependent and ABA-independent signaling are involved in plant responses to stress (Roychoudhury et al., 2013; Yoshida et al., 2014). For example, in *Arabidopsis*, high temperature inhibited the function of the ubiquitin proteasome system, but the inhibition was reduced in ABA biosynthetic mutants and in plants by the treatment of fluridone (ABA biosynthesis inhibitor), suggesting that it was regulated by high temperature in an ABA-dependent manner (Chiu et al., 2016). In *Physcomitrella patens*, the accumulation of low-molecular-weight soluble (LMS) sugars had no significant effect on the ABA-deficient mutant *ppaba1* and its wild type after hyperosmotic and ABA treatments, suggesting that LMS sugars were regulated by hyperosmotic stress in an ABA-independent way (Takezawa et al., 2015).

When maize plants are subjected to adverse conditions, various mechanisms are evoked to deal with stress challenges, which include antioxidant capabilities, osmotic adjustment, photosynthetic rate reduction, and ABA accumulation (Gong et al., 2014; Sah et al., 2016). These processes involve the expression of stress-response genes, many of which are regulated by ABA (Fan et al., 2016). Therefore, ABA as an important messenger regulates the adaptive response of plants to abiotic stress (Sah et al., 2016). Currently, although the identification of ABA receptors has greatly increased our understanding of ABA perception in plants, this is not enough to identify the proteins regulated by ABA when maize plants are subjected to drought stress.

For this reason, this study aimed to better identify the key proteins regulated by ABA when maize seedlings were exposed to drought stress, and ultimately use this information to guide agricultural planning and minimize maize yield reduction caused by drought stress. Maize mutant *viviparous*-*5* (*vp5*) is deficient in ABA biosynthesis and suffers photo-bleaching of leaves under normal light conditions (Robichaud et al., 1980; Hable et al., 1998), and has a much reduced ABA content in maize plants compared with wild-type *Vp5* plants (Hu et al., 2015). Thus, the mutant *vp5* and its wild-type *Vp5* are ideal materials in which to identify key drought-response proteins regulated by ABA in maize plants. In this study, the differentially expressed proteins in maize plants exposed to drought stress were identified by multiplex run iTRAQ-based quantitative proteomic analysis and liquid chromatography-tandem mass spectrometry (LC-MS/MS) methods. As a result, 150 proteins with significant changes in expression level were identified as being significantly regulated by drought stress in an ABA-dependent or -independent way.

## Methods

### Plant material and treatments

Maize mutant *vp5* and its wild-type *Vp5* seedlings were used in the present study. As described previously (Hu et al., 2015), the *vp5* mutant has reduced amounts of ABA because of a deficiency in ABA biosynthesis (Robichaud et al., 1980). Homozygous recessive kernels (*vp5*/*vp5*) lack carotenoids, causing white endosperm and embryos, which are easily distinguishable from the yellow wild-type kernels (*Vp5*/–). Because the recessive mutation is lethal in the homozygous state, it is maintained as a heterozygote. Seeds of *vp5* and *Vp5* plants were obtained by selfing plants grown from heterozygous seeds (Maize Genetics Stock Center, Urbana, IL, USA).

According to our previous description (Hu et al., 2015), *Vp5* and *vp5* seeds were germinated on moistened filter paper after being surface-sterilized for 10 min in 2% hypochlorite and then rinsed in distilled water. After germination for 2 days, both *vp5* and *Vp5* seedlings were cultured in Hoagland's nutrient solution in a light chamber (day 28°C/night 22°C, relative humidity 75%) under 400 μmol/m^2^/s photosynthetically active radiation with a 14/10 h (day/night) cycle. After 2 weeks, the seedlings were subjected to drought stress by placing them in a −0.7 MPa PEG6000 solution for 8 h at 28°C under relative humidity of 40%. Control seedlings were maintained at 28°C under relative humidity of 75%. Subsequently, leaves of treated and untreated seedlings were sampled, immediately frozen in liquid N_2_, and stored at −80°C until analysis. Three or five replicates were performed for each treatment.

### The quantum efficiency of photosystem II (ΦPSII)

ΦPSII was measured by using an OS-30p Chlorophyll Fluorometer (Opti-Sciences, Tyngsboro, Massachusetts, USA) in the second full expand leaf.

### Malondialdehyde (MDA)

MDA contents were measured according to a previous description (Hodges et al., 1999). Fifty milligrams fresh weight (FW) leaves in 1 ml 80% (v/v) ethanol were homogenized in a mortar. After centrifugation, the supernatant was reacted with thiobarbituric acid to produce the pinkish-red chromogen, thiobarbituric acid-malondialdehyde. Absorbance was measured at 440, 532, and 600 nm by using a UV-vis spectrophotometer. MDA content was calculated as nmol/g FW tissue.

### Protein extraction

According to our previous description (Hu et al., 2015), total proteins from the second newly expanded leaf of the maize seedlings were extracted according to the following procedure. Approximately 0.5 g of fresh leaves from each biological replicate were ground into a fine power in liquid N_2_ in a mortar and further ground in 4 ml SDS buffer (30% sucrose, 2% SDS, 100 mM Tris-HCl, pH 8.0, 50 mM EDTA-Na_2_, 20 mM DTT) and 4 ml phenol (Tris-buffered, pH 8.0) in a 10 ml tube, followed by the addition of 1 mM phenylmethanesulfonyl fluoride and PhosSTOP Phosphatase Inhibitor Cocktail (one tablet/10 ml; Roche, Basel, Switzerland) to inhibit protease and phosphatase activity, respectively. The mixture was thoroughly vortexed for 30 s, and the phenol phase was separated by centrifugation at 14,000 × g and 4°C for 15 min. The upper phenol phase was pipetted into fresh 10 ml tubes, and 4-fold volumes of cold methanol plus 100 mM ammonium acetate were added. After centrifugation at 14,000 × g and 4 °C for 15 min, the supernatant was carefully discarded and the precipitated proteins were washed twice with cold acetone. Finally, the protein mixtures were harvested by centrifugation. Measurement of protein content was carried out using a 2-D Quant Kit (Amersham Bioscience, America) containing bovine serum albumin (2 mg/ml) as the standard. To enhance the quantitative accuracy, proteins extracted from every biological replicate were adjusted to the same concentration for the subsequent analysis (Wang et al., 2013; Hu et al., 2015; Zhang et al., 2015).

### Protein digestion and iTRAQ labeling

As described previously (Hu et al., 2015), protein digestion was performed according to the FASP procedure (Umezawa et al., 2013), and the resulting peptide mixture was labeled using the 4-plex iTRAQ reagent according to the manufacturer's instructions (Applied Biosystems). Briefly, 200 μg proteins for each sample were incorporated into 30 μl STD buffer (4% SDS, 100 mM DTT, 150 mM Tris-HCl, pH 8.0). The detergent DTT and other low-molecular-weight components were removed using UA buffer (8 M urea, 150 mM Tris-HCl, pH 8.0) by repeated ultrafiltration (Microcon units, 30 kD). Then, 100 μl of 0.05 M iodoacetamide in UA buffer was added to block reduced cysteine residues, and the samples were incubated for 20 min in darkness. The filters were washed with 100 μl UA buffer three times and then washed twice with 100 μl DS buffer (50 mM trimethylammonium bicarbonate at pH 8.5). Finally, the protein suspensions were digested with 2 μg trypsin (Promega) in 40 μl DS buffer overnight at 37°C, and the resulting peptides were collected as a filtrate. The peptide content was estimated by UV light spectral density at 280 nm of a 0.1% solution using an extinction coefficient of 1.1 that was calculated on the basis of the frequency of tryptophan and tyrosine in vertebrate proteins.

For labeling, each iTRAQ reagent was dissolved in 70 μl ethanol and added to the respective peptide mixture. The samples, *Vp5*-control, *Vp5*-drought, *vp5*-control, and *vp5*-drought, were multiplexed and vacuum dried. Three independent biological experiments were performed.

### Peptide fractionation with strong cation exchange chromatography

According to our previous description (Hu et al., 2015), iTRAQ-labeled peptides were fractionated by strong cation exchange chromatography using the AKTA Purifier system (GE Healthcare). The dried peptide mixture was reconstituted and acidified with 2 ml buffer A (10 mM KH_2_PO_4_ in 25% ACN, pH 2.7) and loaded onto a PolySULFOETHYL 4.6 × 100 mM column (5 μm, 200 Å, PolyLC Inc, Maryland, USA). The peptides were eluted at a flow rate of 1 ml/min with a gradient of 0–10% buffer B (500 mM KCl, 10 mM KH_2_PO_4_ in 25% of ACN, pH 2.7) for 2 min, 10–20% buffer B for 25 min, 20–45% buffer B for 5 min, and 50–100% buffer B for 5 min. The elution was monitored by absorbance at 214 nm, and fractions were collected every 1 min. The collected fractions (about 30 fractions) were finally combined into 10 pools and desalted on C18 Cartridges [Empore™ SPE Cartridges C18 (standard density), bed inner diameter 7 mm, volume 3 ml, Sigma]. Each fraction was concentrated by vacuum centrifugation and reconstituted in 40 μl 0.1% (v/v) trifluoroacetic acid. All samples were stored at −80°C until LC-MS/MS analysis.

### Liquid chromatography (LC)-electrospray ionization (ESI) tandem MS (MS/MS) analysis by Q exactive

As described previously (Hu et al., 2015), experiments were performed on a Q Exactive mass spectrometer that was coupled to an Easy nLC (Proxeon Biosystems, now Thermo Fisher Scientific). Ten microliters of each fraction were injected for nanoLC-MS/MS analysis. The peptide mixture (5 μg) was loaded onto a C18 reversed-phase column (Thermo Scientific Easy Column, 10 cm long, 75 μm inner diameter, 3 μm resin) in buffer A (0.1% formic acid) and separated with a linear gradient of buffer B (80% acetonitrile and 0.1% formic acid) at a flow rate of 250 nl/min controlled by IntelliFlow technology over 140 min. MS data were acquired using a data-dependent top10 method dynamically choosing the most abundant precursor ions from the survey scan (300–1800 m/z) for higher-energy collisional dissociation fragmentation. Determination of the target value was based on predictive automatic gain control. Dynamic exclusion duration was 60 s. Survey scans were acquired at a resolution of 70,000 at m/z 200, and the resolution for higher-energy collisional dissociation spectra was set to 17,500 at m/z 200. Normalized collision energy was 30 eV, and the underfill ratio, which specifies the minimum percentage of the target value likely to be reached at maximum fill time, was defined as 0.1%. The instrument was run with peptide recognition mode enabled.

### Data analysis

According to our previous description (Hu et al., 2015), MS/MS spectra were searched using Mascot 2.2 (Matrix Science) embedded in Proteome Discoverer 1.4 against the uniprot_Zea_mays_87227_20150504.fasta (87227 sequences, download May 4th, 2015) and the decoy database. The parameters used in Mascot searches for normal peptides were as follows: Peptide mass tolerance: 20 ppm, MS/MS tolerance: 0.1 Da, Enzyme: Trypsin, max missed cleavage: 2, Fixed modification: Carbamidomethyl (C), iTRAQ4plex(K), iTRAQ4plex(N-term), Variable modification:Oxidation (M), FDR ≤ 0.01. The protein and peptide probabilities were set at 50 and 60%, respectively. Proteins with at least two unique peptides, a Mascot score of at least 25, and detection in at least two replicates were further used.

For each replicate of proteomics, iTRAQ ratios between drought stressed plants and controls for each run were converted to *z*-scores to normalize the data.

### Bioinformatics

As described previously (Hu et al., 2015), the molecular functions of the identified proteins were classified according to their gene ontology annotations and their biological functions. The subcellular localization of the unique proteins identified in this study was predicted using the publicly available program WolfPsort (http://wolfpsort.org). Protein-protein interaction networks were analyzed using the publicly available program STRING (http://string-db.org/). STRING is a database of known and predicted protein-protein interactions. The interactions include direct (physical) and indirect (functional) associations, and they are derived from four sources: the genomic context, high-throughput experiments, coexpression, and previous knowledge. STRING quantitatively integrates the interaction data from these sources for a large number of organisms and, where applicable, transfers information between these organisms.

### ABA assay

According to our previous description (Hu et al., 2012), maize leaves (0.5–1.0 g) were ground in liquid N_2_ with a mortar, extracted with 2 ml of ice-cold 80% methanol containing 1 mM butylated hydroxytoluene to prevent oxidation, and then stored overnight at 4°C. The extracts were centrifuged at 12,000 × g for 15 min at 4°C. The pellets were extracted once and stored at 4°C for 1 h. The two resulting supernatants were combined and passed through a C18 Sep-Pak cartridge (Waters, Milford, MA, USA). The efflux was collected and dried in N_2_. The residues were then dissolved in 10 mM phosphate buffer solution (pH 7.4), and concentrations of ABA were determined in enzyme-linked immunosorbent assay (ELISA). Statistical analyses of the physiological measurements were conducted using independent Student's *t*-tests with SPSS statistics software (version 17.0).

### Gene expression analysis by reverse-transcription PCR

As described previously (Hu et al., 2010), total RNA was isolated from leaves by using an RNeasy mini kit according to the instructions supplied by the manufacturer (Qiagen, Valencia, CA). Approximately 3 μg of total RNA was reverse transcribed into cDNA using SuperScript II reverse transcriptase (Invitrogen, Carlsbad, CA). cDNA was amplified by PCR using the primers shown in Supplementary Table S1. To standardize the results, the relative abundance of β-actin was also determined and used as the internal standard. Aliquots of the PCR reactions were loaded on agarose gels and stained with ethidium bromide.

### Statistical analysis

The proteins and ABA assays were the mean of three replicates. The means were compared by a one-way analysis of variance and Duncan's multiple range test at a 5% level of significance. False discovery rates attained by the Benjamini-Hochberg method were used to adjust *p*-values (correction for multiple comparisons). The significance of differences between *Vp5* and *vp5* were compared by *t*-test analysis at the 5% level.

## Results

### The influence of drought on ABA, MDA, and ΦPSII

After *vp5* and *Vp5* plants were subjected to drought treatment, the ABA content of leaves was measured by ELISA. Whether under normal or drought stress conditions, the ABA content in *Vp5* leaves was more than 6-fold higher than that in *vp5* leaves; compared with the corresponding control, the relative increase in ABA content was 120.45 and 21.98% in *Vp5* and *vp5* leaves under drought stress, respectively (Table 1). MDA is generated by lipid peroxidation, so the change in MDA content reflects the extent of membrane damage. Our results indicated that MDA content was prominently elevated by drought stress, and the relative increase was 193.1 and 232.6% in *Vp5* and *vp5* leaves, respectively, compared with the corresponding control (Table 1). ΦPSII is one of the chlorophyll fluorescence parameters and is classically used to monitor change in photosynthetic performance. The present results indicated that the relative decrease in ΦPSII caused by drought stress was 33.00 and 39.5% in *Vp5* and *vp5* leaves, respectively, compared with the respective controls. Regardless of drought stress level, the ΦPSII of *Vp5* was significantly more than that of *vp5*. These results indicate that the ABA-deficient mutant *vp5* is more susceptible to drought stress than *Vp5* and is very useful material to study the key proteins regulated by ABA under drought stress.

**Table 1:** **ABA and MDA content, and ΦPSII in maize *vp5* and *Vp5* leaves under control and drought conditions**.

	**Gene type**	**Control**	**Drought**	**Relative increase/decrease (%)**
ABA	*Vp*5	76.50 (ng/gFW) b	168.64 (ng/gFW) a	120.45 ↑
	*vp*5	17.28 (ng/gFW) d	21.08 (ng/gFW) c	21.98 ↑
MDA	*Vp*5	5.12 (nmol/gFW) d	15.01 (nmol/gFW) b	193.1 ↑
	*vp*5	7.45 (nmol/gFW) c	24.78 (nmol/gFW) a	232.6 ↑
ΦPSII	*Vp*5	0.612 a	0.410 b	33.00 ↓
	*vp*5	0.210 c	0.128 d	39.05 ↓

Each value represents the average of three biological replicas. For Dun's Results, different characters are considered to be significant among different treatments. The symbols “↑and ↓” represent relative increase and decrease, respectively.

### Identification of differentially expressed proteins under drought stress

**Figure 1:**
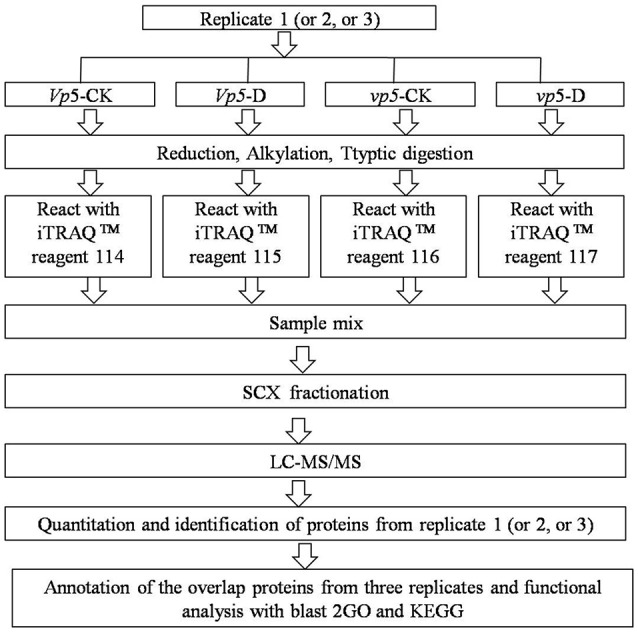
**iTRAQ 4-plex labeling and LC MS/MS workflow for identifying proteins in the ABA-deficient mutant *vp5* and wild-type *Vp5* seedling leaves under drought conditions**.

**Figure 2:**
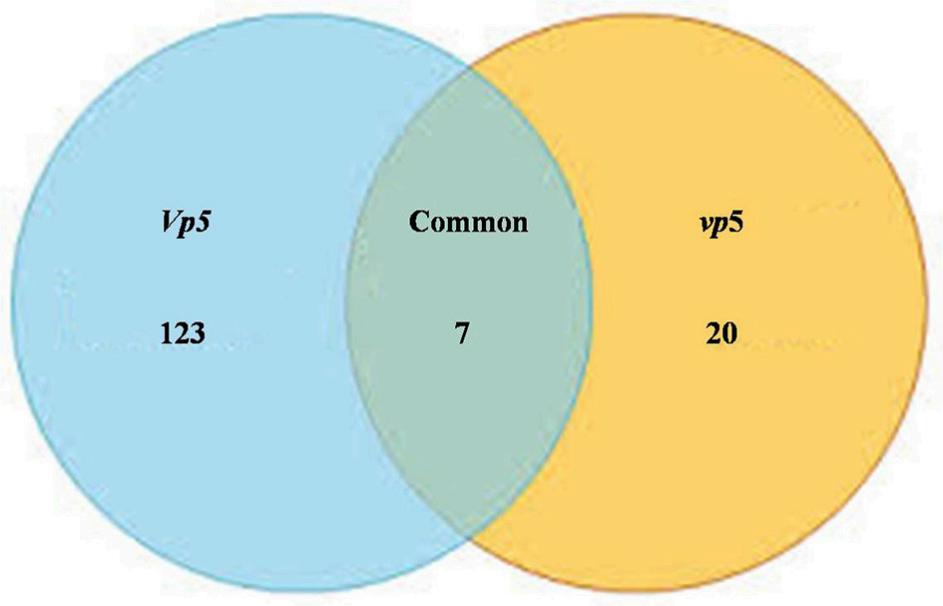
**Venn diagram showing the number of significantly expressed proteins in maize leaves under drought stress**. The diagram shows the overlap between the ABA-deficient mutant *vp5* and the wild-type *Vp5*.

**Figure 3:**
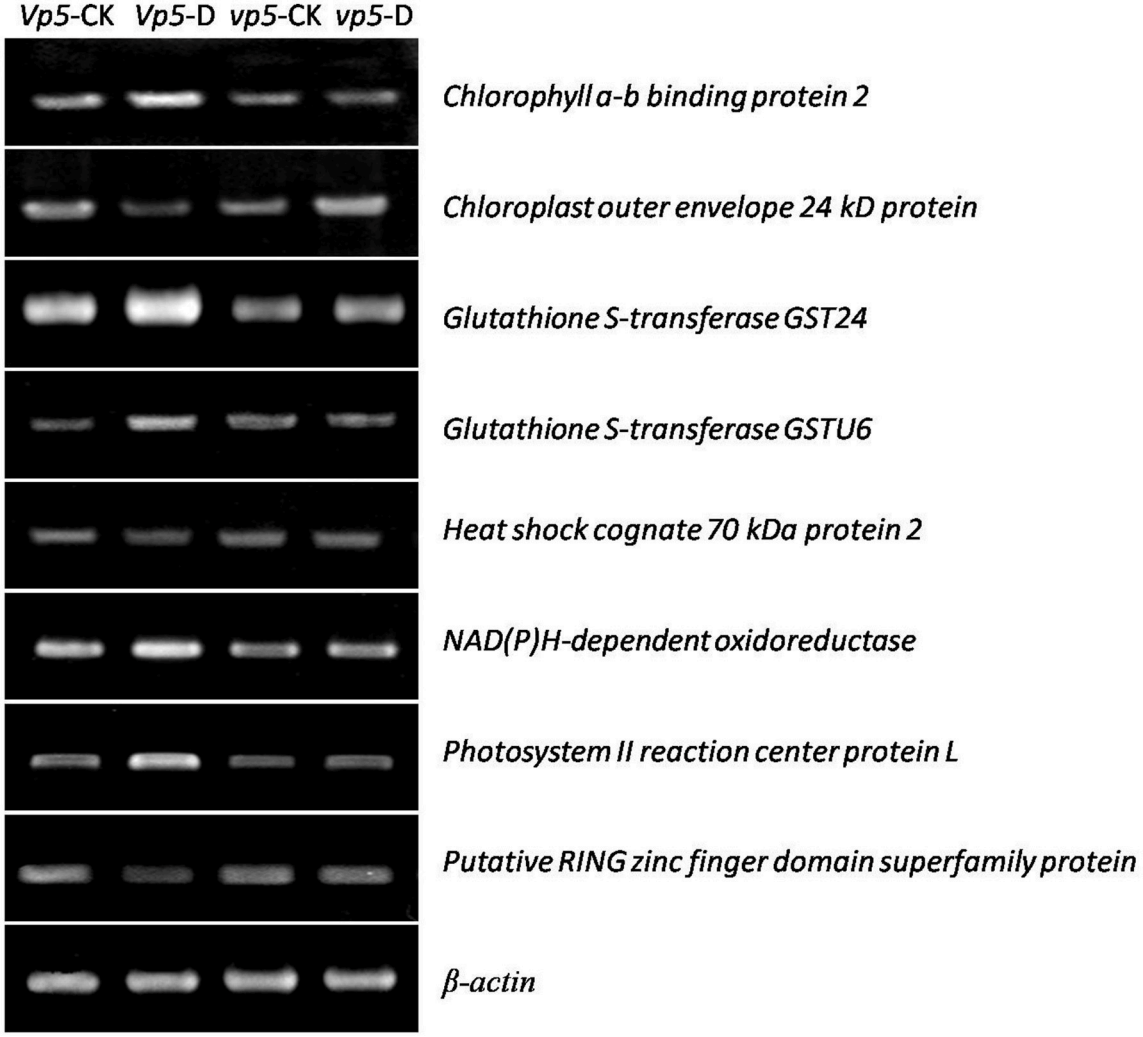
**Gene expression analysis of eight proteins in maize *Vp5* and *vp5* leaves under drought stress conditions**. The eight proteins included chlorophyll a-b binding protein 2, chloroplast outer envelope 24 kD protein, glutathione S-transferase GST 24, glutathione S-transferase GSTU6, heat shock cognate 70 kDa protein 2, NAD(P)H-dependent oxidoreductase, photosystem II reaction center protein L, and putative RING zinc finger domain superfamily protein. Experiments were repeated at least three times.

After *vp5* and *Vp5* seedlings were subjected to drought stress, total proteins from leaves were extracted and analyzed by multiplex run iTRAQ-based quantitative proteomic analysis and LC-MS/MS methods according to the workflow shown in Figure 1, resulting in the identification of 7051 proteins at a false discovery rate of 1%. Of the 7051 proteins (indentified peptides, see Supplementary Excels S1–S3), based on *p* < 0.01, there were 150 proteins with a ≥1.5-fold (increased) or ≤ 0.66-fold (decreased) expression change ratio under drought stress relative to the respective controls (Figure 2). In order to validate the above-mentioned results of protein abundance, the transcript expression levels of eight interesting proteins were analyzed by reverse-transcription PCR. The results indicated that the transcription patterns of the eight proteins were concomitant with protein expression levels, which supported the results attained by iTRAQ-based quantitative proteomic analysis and LC-MS/MS (Figure 3).

**Figure 4:**
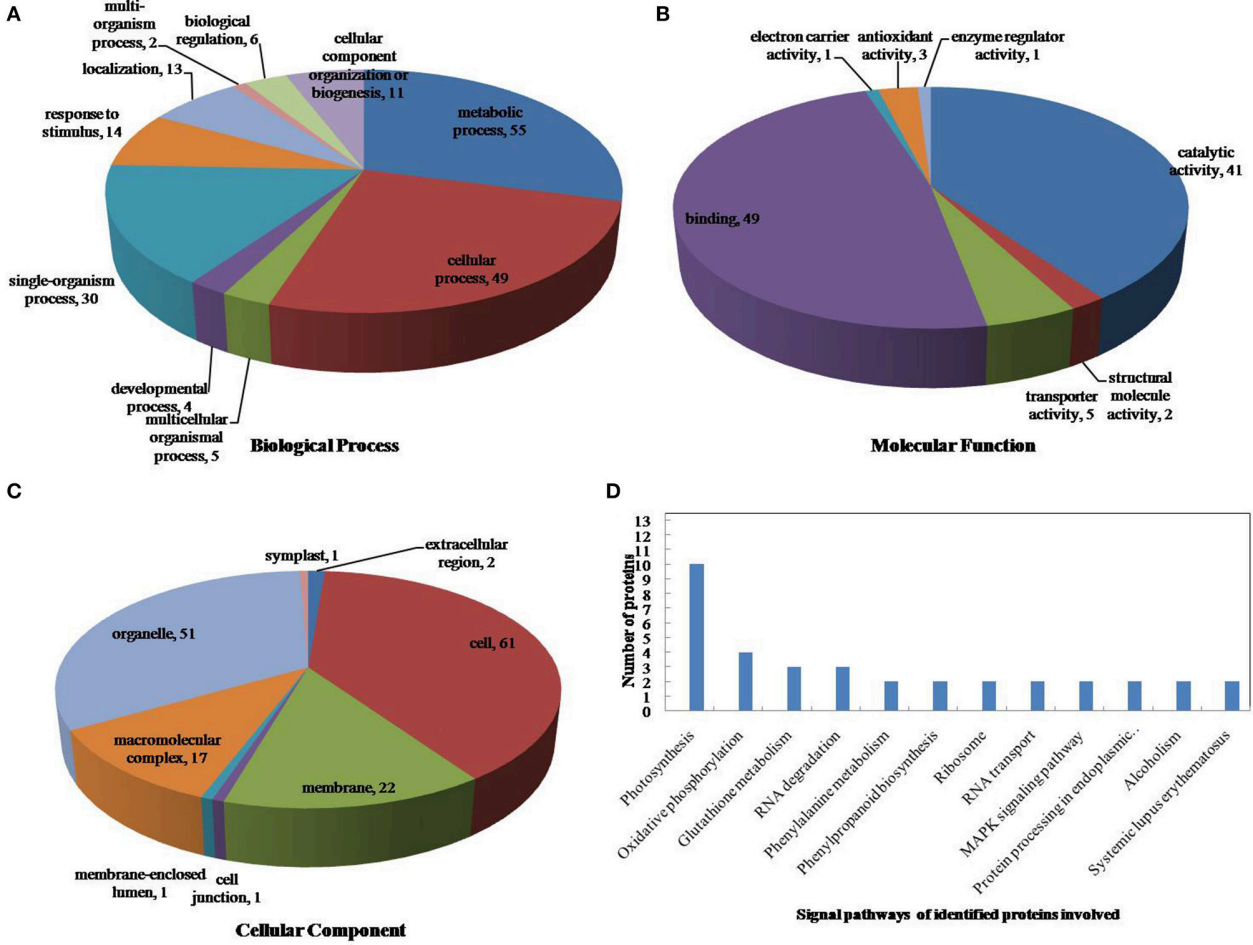
**Pie charts of the distribution of differentially expressed proteins based on their predicted molecular (A)** biological process, **(B)** molecular functions, and **(C)** cellular components, and **(D)** the signaling pathways of the proteins identified in maize *Vp5* leaves subjected to drought stress. In this study, 130 proteins were identified under drought stress and were classified by their known or predicted subcellular localization using Blast2Go (http://www.blast2go.com).

**Figure 5:**
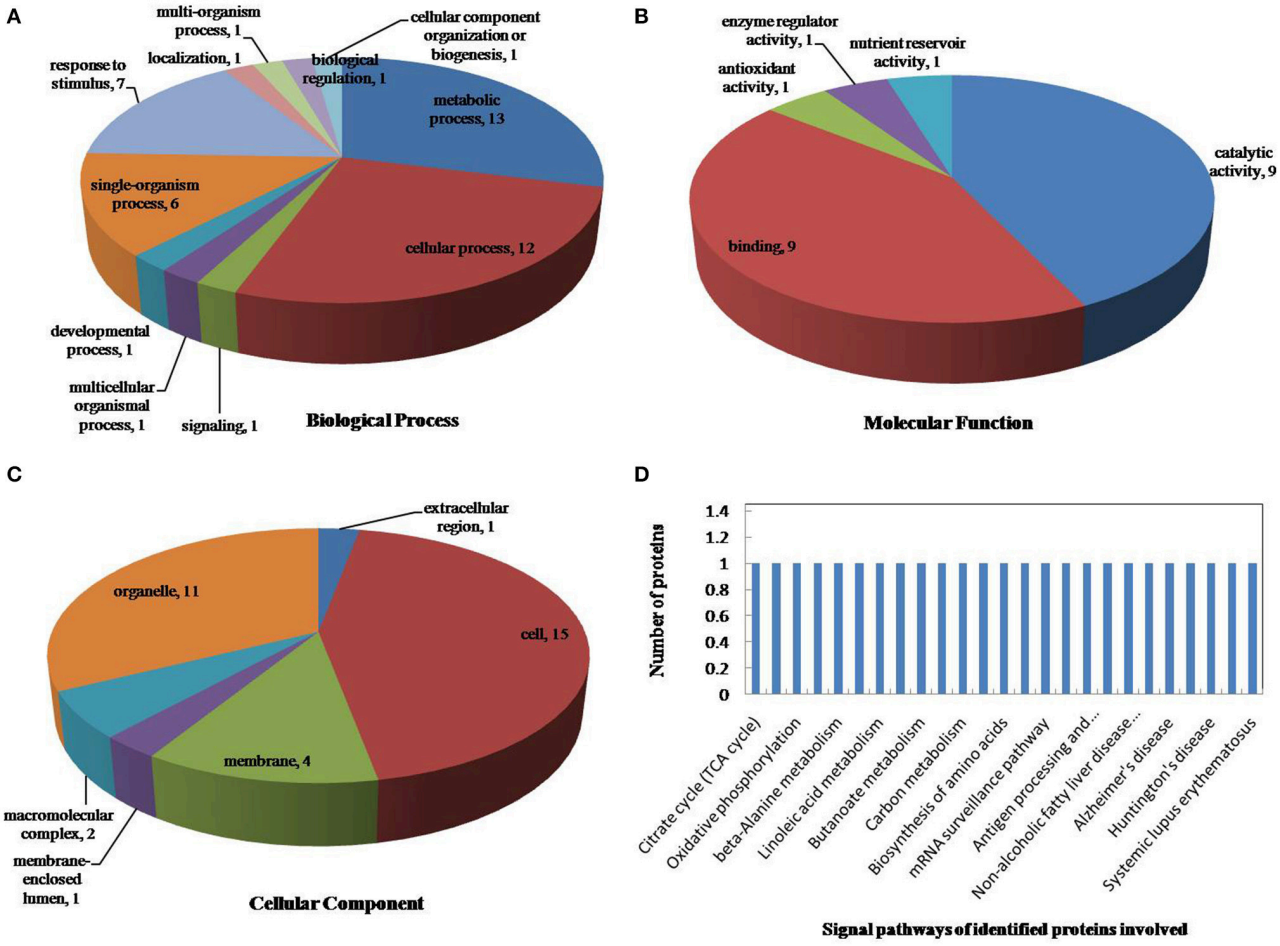
**Pie charts of the distribution of differentially expressed proteins by their predicted (A)** biological process, **(B)** molecular functions, and **(C)** cellular components, and **(D)** the signaling pathways of the proteins identified in maize *Vp5* leaves under drought stress. In this study, 27 proteins were identified under drought stress, and they were classified by their known or predicted cellular localization using Blast2Go (http://www.blast2go.com).

**Figure 6:**
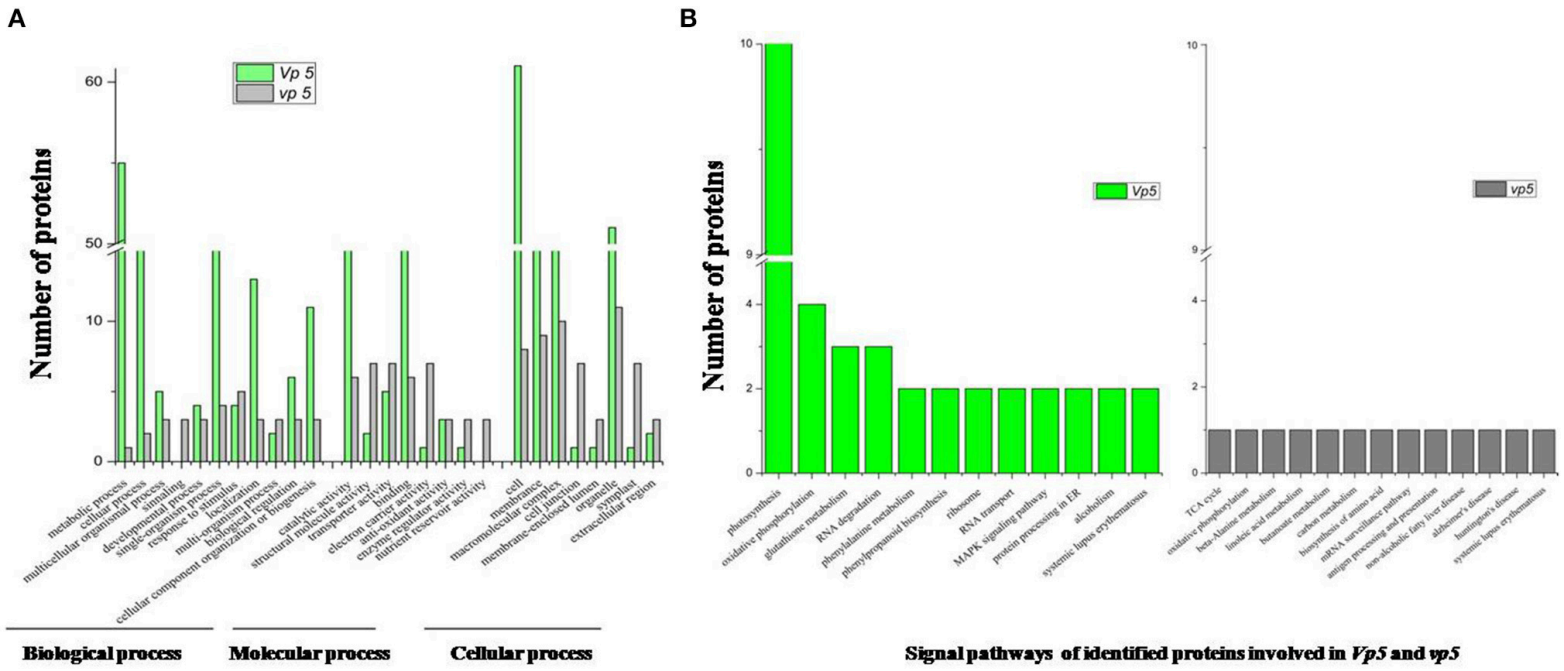
**Comparisons of the (A)** biological process, molecular functions, cellular components, and **(B)** signaling pathways (from Figures 4, 5) of differentially expressed proteins identified in maize *Vp5* and *vp5* leaves under drought stress. The proteins were classified by their known or predicted cellular function using Blast2GO (http://www.blast2go.com).

The drought-response proteins in *vp5* and *Vp5* were annotated using Blast2GO according to the biological process “cell component and molecular function” (Figures 4, 5A–C). The results showed that the differentially expressed proteins in *Vp5* were mainly involved in such signal pathways as photosynthesis, oxidative phosphorylation, glutathione metabolism, and RNA degradation (Figure 4D), which was different from the signaling pathways identified in *vp5* (Figure 5D). Figure 6 shows the comparisons of the biological process, molecular functions, cellular components, and signaling pathways of proteins identified in *Vp5* and *vp5* under drought stress, which clearly indicated a difference in significantly expressed proteins between *Vp5* and *vp5* under drought stress.

### ABA regulation for drought-response proteins

Plants respond to abiotic stress by ABA-dependent and ABA-independent mechanisms (Sah et al., 2016). In the present study, 150 proteins with a fold change ≥1.5 included 7 identified in both genotypes (Table 2), 123 only in *Vp5* (Table 3), and 20 only in *vp5* (Table 4). Specifically, among the 7 (Table 2), 123 (Table 3), and 20 (Table 4) proteins, there were 5, 64, and 8 uncharacterized proteins, respectively. Among the seven common proteins in the two genotypes (Table 2), the expression of four proteins (C0HF37, B7ZX39, B6T3J3, and K7TKJ3) was increased in *Vp5* but decreased in *vp5* under drought conditions, indicating that these proteins were up-regulated by drought stress in an ABA-dependent manner; drought stress decreased the expression of protein B4FMW4 by a similar extent in *Vp5* and *vp5*, indicating that the protein was down-regulated by drought stress in an ABA-independent manner; drought stress significantly decreased the expression of two proteins (B6TM56, C0PBJ1) in *Vp5*, but significantly increased the expression of these two proteins in *vp5*, indicating that they were up-regulated by drought stress but down-regulated by ABA.

**Table 2:** **Proteins with significant expression level changes in both *Vp5* and *vp5* leaves under drought stress**.

**Accession**	**Description**	***Vp5*****: D/control**			***vp5*****: D/control**			***Vp5*****: D/control**		***vp5*****: D/contro**		***T*-test**	**Regulation of ABA and drought stress**
		**1**	**2**	**3**	**1**	**2**	**3**	**Average^a^**	***P*-value**	**Average^a^**	***P*-value**		
C0HF37	Uncharacterized protein	1.620	1.632	1.545	0.504	0.518	0.514	1.599	0.002	0.512	0.001	0.000	Up-regulated by drought in an ABA-dependent way
B4FMW4	Uncharacterized protein	0.601	0.613	0.526	0.546	0.545	0.542	0.580	0.003	0.544	0.001	0.334	Down-regulated by drought in an ABA-independent way
B7ZX39	Uncharacterized protein	1.615	1.627	1.540	0.541	0.552	0.546	1.594	0.001	0.546	0.001	0.000	Up-regulated by drought in an ABA-dependent way
B6T3J3	Histone H2A	1.672	1.691	1.590	0.561	0.575	0.571	1.651	0.001	0.569	0.002	0.000	Up-regulated by drought in an ABA-dependent way
K7TKJ3	Uncharacterized protein	5.551	5.063	5.076	0.621	0.635	0.632	5.230	0.000	0.629	0.003	0.000	Up-regulated by drought in an ABA-dependent way
B6TM56	Chloroplast outer envelope 24 kD protein	0.618	0.630	0.543	1.529	1.543	1.539	0.597	0.003	1.537	0.001	0.000	Up-regulated by drought, but down-regulated by ABA
C0PBJ1	Uncharacterized protein	0.465	0.477	0.390	1.539	1.553	1.548	0.444	0.001	1.547	0.000	0.000	Up-regulated by drought, but down-regulated by ABA

a Each value represents the average of three biological replicates. The average is significant at a p < 0.01 level. A t-test value <0.05 is considered to be significant between ABA-deficient mutant vp5 and wild-type Vp5. D, drought stress.

**Table 3:** **Proteins with significant expression level changes only in *Vp5* leaves under drought stress**.

**Accession**	**Description**	***Vp5*****: OS/control**			***vp5*****: OS/control**			***Vp5*****: OS/control**		***vp5*****: OS/contro**		***T*-test**	**Regulation of ABA and drought stress**
		**1**	**2**	**3**	**1**	**2**	**3**	**Average^a^**	***P*-value**	**Average^a^**	***P*-value**		
B4F9L3	Carbonic anhydrase	0.627	0.592	0.602	0.970	0.962	0.984	0.607	0.003	0.972	0.655	0.000	Down-regulated by drought in an ABA-dependent way
B4F9Q3	Uncharacterized protein	1.825	1.890	1.850	0.916	0.898	0.910	1.855	0.000	0.908	0.188	0.000	Up-regulated by drought in an ABA-dependent way
B4F9U4	Putative RING zinc finger domain superfamily protein	0.534	0.499	0.509	1.084	1.076	1.098	0.514	0.001	1.086	0.213	0.000	Down-regulated by drought in an ABA-dependent way
B4FA94	Uncharacterized protein	0.537	0.502	0.512	1.037	1.049	1.031	0.517	0.001	1.039	0.538	0.000	Down-regulated by drought in an ABA-dependent way
B4FB53	Uncharacterized protein	1.527	1.492	1.502	1.060	1.052	1.074	1.507	0.001	1.062	0.346	0.000	Up-regulated by drought in an ABA-dependent way
B4FB57	Uncharacterized protein	2.096	2.001	2.131	1.031	1.023	1.045	2.076	0.000	1.033	0.600	0.000	Up-regulated by drought in an ABA-dependent way
B4FB96	Uncharacterized protein	0.661	0.626	0.636	0.979	0.971	0.993	0.641	0.004	0.981	0.760	0.000	Down-regulated by drought in an ABA-dependent way
B4FBH1	Uncharacterized protein	0.677	0.642	0.652	1.015	1.007	1.029	0.657	0.004	1.017	0.784	0.000	Down-regulated by drought in an ABA-dependent way
B4FBV4	Chaperone protein dnaJ 10 isoform 1	1.705	1.793	1.740	0.934	0.926	0.948	1.746	0.000	0.936	0.332	0.000	Up-regulated by drought in an ABA-dependent way
B4FBY1	Chaperone protein dnaJ	1.646	1.611	1.621	0.956	0.928	0.930	1.626	0.000	0.938	0.349	0.000	Up-regulated by drought in an ABA-dependent way
B4FDK8	Uncharacterized protein	1.593	1.558	1.568	1.057	1.049	1.071	1.573	0.001	1.059	0.367	0.000	Up-regulated by drought in an ABA-dependent way
B4FDY7	Eukaryotic translation initiation factor 3 subunit E	1.631	1.596	1.606	1.249	1.241	1.263	1.611	0.000	1.251	0.001	0.000	Up-regulated by drought in an ABA-dependent way
B4FEE1	Uncharacterized protein	1.523	1.488	1.498	1.256	1.248	1.270	1.503	0.001	1.258	0.001	0.000	Up-regulated by drought in an ABA-dependent way
B4FFW5	Uncharacterized protein	1.823	1.848	1.828	0.949	1.041	0.863	1.833	0.000	0.951	0.561	0.000	Up-regulated by drought in an ABA-dependent way
B4FI24	Uncharacterized protein	0.681	0.646	0.656	1.275	1.167	1.089	0.661	0.004	1.177	0.009	0.001	Down-regulated by drought in an ABA-dependent way
B4FJ71	Uncharacterized protein	0.577	0.542	0.552	1.020	1.012	1.034	0.557	0.002	1.022	0.724	0.000	Down-regulated by drought in an ABA-dependent way
B4FJH1	Uncharacterized protein	0.500	0.465	0.475	1.024	1.016	1.038	0.480	0.001	1.026	0.678	0.000	Down-regulated by drought in an ABA-dependent way
B4FK45	Uncharacterized protein	1.581	1.546	1.556	0.890	0.882	0.904	1.561	0.001	0.892	0.137	0.000	Up-regulated by drought in an ABA-dependent way
B4FP86	Uncharacterized protein	2.291	2.456	2.366	0.999	0.991	1.013	2.371	0.000	1.001	0.987	0.000	Up-regulated by drought in an ABA-dependent way
B4FPH3	Uncharacterized protein	1.610	1.575	1.585	1.028	1.020	1.042	1.590	0.001	1.030	0.633	0.000	Up-regulated by drought in an ABA-dependent way
B4FPM5	Uncharacterized protein	1.814	1.789	1.809	1.017	1.009	1.031	1.804	0.000	1.019	0.760	0.000	Up-regulated by drought in an ABA-dependent way
B4FPS3	Uncharacterized protein	1.637	1.602	1.612	0.900	0.862	0.854	1.617	0.000	0.872	0.018	0.000	Up-regulated by drought in an ABA-dependent way
B4FQN6	Sorbitol transporter	0.682	0.647	0.657	1.332	1.324	1.346	0.662	0.005	1.334	0.005	0.000	Down-regulated by drought in an ABA-dependent way
B4FRG9	Uncharacterized protein	0.620	0.585	0.595	0.952	0.944	0.966	0.600	0.002	0.954	0.473	0.000	Down-regulated by drought in an ABA-dependent way
B4FWT5	Soluble inorganic pyrophosphatase	1.552	1.517	1.527	0.942	0.934	0.956	1.532	0.001	0.944	0.390	0.000	Up-regulated by drought in an ABA-dependent way
B4FX06	Uncharacterized protein	0.568	0.533	0.543	0.909	0.901	0.923	0.548	0.002	0.911	0.200	0.000	Down-regulated by drought in an ABA-dependent way
B4FX77	Uncharacterized protein	0.679	0.644	0.654	1.213	1.005	1.127	0.659	0.004	1.115	0.241	0.002	Down-regulated by drought in an ABA-dependent way
B4FZ22	Uncharacterized protein	0.681	0.646	0.656	1.031	0.923	0.845	0.661	0.004	0.933	0.444	0.008	Down-regulated by drought in an ABA-dependent way
B4G1A3	Uncharacterized protein	1.533	1.498	1.508	0.903	0.895	0.917	1.513	0.001	0.905	0.177	0.000	Up-regulated by drought in an ABA-dependent way
B4G1H1	Uncharacterized protein	1.599	1.564	1.574	1.295	1.287	1.309	1.579	0.001	1.297	0.007	0.000	Up-regulated by drought in an ABA-dependent way
B6SHW9	Ubiquitin fusion protein	0.668	0.633	0.643	1.059	1.051	1.073	0.648	0.004	1.061	0.353	0.000	Down-regulated by drought in an ABA-dependent way
B6SJL2	Thaumatin-like protein	0.649	0.614	0.624	0.958	0.950	0.972	0.629	0.003	0.960	0.529	0.000	Down-regulated by drought in an ABA-dependent way
B6SK13	Ribose-phosphate pyrophosphokinase 4	1.888	1.853	1.863	0.921	0.913	0.935	1.868	0.000	0.923	0.256	0.000	Up-regulated by drought in an ABA-dependent way
B6SMB0	Cytochrome b-c1 complex subunit 6	0.675	0.640	0.650	0.991	0.983	1.005	0.655	0.004	0.993	0.910	0.000	Down-regulated by drought in an ABA-dependent way
B6SP61	Ferredoxin-1	0.655	0.620	0.630	0.762	0.754	0.776	0.635	0.003	0.764	0.015	0.000	Down-regulated by drought in an ABA-dependent way
B6SSB3	Anthocyanidin 5,3-O-glucosyltransferase	1.915	1.880	1.890	0.821	0.813	0.835	1.895	0.000	0.823	0.004	0.000	Up-regulated by drought in an ABA-dependent way
B6STN4	Chlorophyll a-b binding protein 2	1.641	1.606	1.616	0.861	0.853	0.875	1.621	0.000	0.863	0.008	0.000	Up-regulated by drought in an ABA-dependent way
B6SU20	Tubby-like protein	1.674	1.639	1.649	1.166	1.158	1.180	1.654	0.000	1.168	0.044	0.000	Up-regulated by drought in an ABA-dependent way
B6SU31	Glutathione peroxidase	0.613	0.578	0.588	0.986	0.978	1.000	0.593	0.002	0.988	0.846	0.000	Down-regulated by drought in an ABA-dependent way
B6SV53	Copper transporter 1	0.670	0.635	0.645	1.109	1.101	1.123	0.650	0.004	1.111	0.129	0.000	Down-regulated by drought in an ABA-dependent way
B6SXR2	Photosystem I reaction center subunit XI	1.604	1.569	1.579	0.837	0.829	0.851	1.584	0.001	0.839	0.005	0.000	Up-regulated by drought in an ABA-dependent way
B6SZ69	Heat shock cognate 70 kDa protein 2	0.674	0.639	0.649	0.868	0.860	0.882	0.654	0.004	0.870	0.019	0.000	Down-regulated by drought in an ABA-dependent way
B6SZK3	NAD(P)H-dependent oxidoreductase	1.857	1.822	1.832	0.933	0.925	0.947	1.837	0.000	0.935	0.326	0.000	Up-regulated by drought in an ABA-dependent way
B6SZL9	Leucine-rich repeat receptor protein kinase EXS	1.559	1.524	1.534	1.066	1.058	1.080	1.539	0.001	1.068	0.307	0.000	Up-regulated by drought in an ABA-dependent way
B6T022	Bowman-Birk type trypsin inhibitor	1.694	1.659	1.669	0.833	0.825	0.847	1.674	0.000	0.835	0.047	0.000	Up-regulated by drought in an ABA-dependent way
B6T072	Putative uncharacterized protein	0.586	0.551	0.561	0.881	0.863	0.905	0.566	0.002	0.883	0.043	0.000	Down-regulated by drought in an ABA-dependent way
B6T1H8	B12D protein	0.584	0.549	0.559	1.011	1.003	1.025	0.564	0.001	1.013	0.683	0.000	Down-regulated by drought in an ABA-dependent way
B6T1Q2	4F5 protein family protein	1.602	1.567	1.577	1.087	1.079	1.101	1.582	0.000	1.089	0.180	0.000	Up-regulated by drought in an ABA-dependent way
B6T531	Ribonucleoprotein A	0.618	0.583	0.593	0.748	0.740	0.762	0.598	0.001	0.750	0.004	0.000	Down-regulated by drought in an ABA-dependent way
B6T763	Exosome complex exonuclease RRP41	0.376	0.341	0.351	1.079	1.071	1.093	0.356	0.000	1.081	0.222	0.000	Down-regulated by drought in an ABA-dependent way
B6T8S7	Tonneau 1b	0.622	0.587	0.597	0.916	1.108	1.030	0.602	0.001	1.018	0.986	0.002	Down-regulated by drought in an ABA-dependent way
B6TGG7	3-oxoacyl-[acyl-carrier-protein] synthase	0.624	0.589	0.599	1.355	1.347	1.369	0.604	0.001	1.357	0.002	0.000	Down-regulated by drought in an ABA-dependent way
B6TGS2	Fb27	1.513	1.508	1.548	1.312	1.304	1.326	1.523	0.000	1.314	0.003	0.000	Up-regulated by drought in an ABA-dependent way
B6TLU5	Lipid binding protein	1.605	1.570	1.580	0.703	0.695	0.717	1.585	0.000	0.705	0.002	0.000	Up-regulated by drought in an ABA-dependent way
B6TP77	Glutathione S-transferase GSTU6	1.531	1.496	1.506	0.987	0.979	1.001	1.511	0.000	0.989	0.568	0.000	Up-regulated by drought in an ABA-dependent way
B6TRQ5	Tesmin/TSO1-like CXC domain containing protein	0.513	0.478	0.488	0.970	0.962	0.984	0.493	0.000	0.972	0.373	0.000	Down-regulated by drought in an ABA-dependent way
B6TUT9	Putative uncharacterized protein	1.643	1.608	1.618	0.905	0.897	0.919	1.623	0.000	0.907	0.070	0.000	Up-regulated by drought in an ABA-dependent way
B6TVU2	Prefoldin subunit 5	2.406	2.421	2.481	0.999	0.991	1.013	2.436	0.000	1.001	0.743	0.000	Up-regulated by drought in an ABA-dependent way
B6TWU1	Putative uncharacterized protein	0.662	0.627	0.637	1.064	1.056	1.078	0.642	0.001	1.066	0.330	0.000	Down-regulated by drought in an ABA-dependent way
B6TYB4	Aldo-keto reductase yakc	1.692	1.657	1.667	0.695	0.687	0.709	1.672	0.000	0.697	0.002	0.000	Up-regulated by drought in an ABA-dependent way
B6TYM4	C2 domain containing protein	0.650	0.615	0.625	1.121	1.113	1.135	0.630	0.001	1.123	0.075	0.000	Down-regulated by drought in an ABA-dependent way
B6U118	T-complex protein 1 subunit zeta	3.829	3.894	4.004	0.957	0.949	0.971	3.909	0.000	0.959	0.265	0.000	Up-regulated by drought in an ABA-dependent way
B6U4B9	Dual specificity protein phosphatase 4	1.605	1.620	1.680	1.059	1.051	1.073	1.635	0.000	1.061	0.376	0.000	Up-regulated by drought in an ABA-dependent way
B6U5V4	ABA-induced protein	0.341	0.306	0.316	0.905	0.897	0.919	0.321	0.000	0.907	0.070	0.000	Down-regulated by drought in an ABA-dependent way
B6U7X1	Maf-like protein CV_0124	1.567	1.572	1.562	0.935	0.927	0.949	1.567	0.000	0.937	0.148	0.000	Up-regulated by drought in an ABA-dependent way
B6UAH3	Putative uncharacterized protein	0.646	0.611	0.621	1.038	1.030	1.052	0.626	0.001	1.040	0.628	0.000	Down-regulated by drought in an ABA-dependent way
B6UEB0	Lipid binding protein	0.678	0.643	0.653	0.915	0.907	0.929	0.658	0.001	0.917	0.089	0.000	Down-regulated by drought in an ABA-dependent way
B6UG46	Peroxin Pex14	0.649	0.614	0.624	1.328	1.020	1.042	0.629	0.001	1.130	0.036	0.007	Down-regulated by drought in an ABA-dependent way
B7U627	DHN2-like protein	1.776	1.741	1.751	1.147	1.139	1.161	1.756	0.000	1.149	0.041	0.000	Up-regulated by drought in an ABA-dependent way
B8A1T1	Peroxidase	0.666	0.631	0.641	1.055	1.047	1.069	0.646	0.001	1.057	0.417	0.000	Down-regulated by drought in an ABA-dependent way
B8A310	Uncharacterized protein	1.898	2.063	1.973	1.096	1.088	1.110	1.978	0.000	1.098	0.142	0.000	Up-regulated by drought in an ABA-dependent way
C0HEI0	Uncharacterized protein	0.398	0.363	0.373	1.264	1.256	1.278	0.378	0.000	1.266	0.005	0.000	Down-regulated by drought in an ABA-dependent way
C0HFZ5	Uncharacterized protein	1.539	1.504	1.514	0.805	0.797	0.819	1.519	0.000	0.807	0.009	0.000	Up-regulated by drought in an ABA-dependent way
C0HG70	Uncharacterized protein	1.580	1.545	1.555	0.858	0.850	0.872	1.560	0.000	0.860	0.025	0.000	Up-regulated by drought in an ABA-dependent way
C0HGT5	T-complex protein 1 subunit delta	1.536	1.501	1.511	1.214	1.006	1.128	1.516	0.000	1.116	0.255	0.003	Up-regulated by drought in an ABA-dependent way
C0HHB1	Carboxypeptidase	2.804	2.829	2.809	0.934	1.026	0.848	2.814	0.000	0.936	0.299	0.000	Up-regulated by drought in an ABA-dependent way
C0HJ24	Uncharacterized protein	1.608	1.573	1.583	1.046	1.038	1.060	1.588	0.000	1.048	0.521	0.000	Up-regulated by drought in an ABA-dependent way
C0P2V2	Uncharacterized protein	1.621	1.586	1.596	0.925	0.917	0.939	1.601	0.000	0.927	0.115	0.000	Up-regulated by drought in an ABA-dependent way
C0P2V7	Uncharacterized protein	1.534	1.499	1.509	1.046	1.038	1.060	1.514	0.000	1.048	0.521	0.000	Up-regulated by drought in an ABA-dependent way
C0P3Y3	Uncharacterized protein	0.682	0.647	0.657	1.037	1.029	1.051	0.662	0.001	1.039	0.643	0.000	Down-regulated by drought in an ABA-dependent way
C0P5M7	Uncharacterized protein	0.684	0.649	0.659	0.937	0.929	0.951	0.664	0.001	0.939	0.156	0.000	Down-regulated by drought in an ABA-dependent way
C0P727	Uncharacterized protein	0.674	0.639	0.649	1.076	1.068	1.090	0.654	0.001	1.078	0.241	0.000	Down-regulated by drought in an ABA-dependent way
C0PF36	Uncharacterized protein	0.648	0.613	0.623	0.990	0.982	1.004	0.628	0.001	0.992	0.609	0.000	Down-regulated by drought in an ABA-dependent way
C0PFW9	Uncharacterized protein	1.539	1.544	1.534	0.974	0.966	0.988	1.539	0.000	0.976	0.413	0.000	Up-regulated by drought in an ABA-dependent way
C0PIW4	Uncharacterized protein	0.656	0.621	0.631	1.001	0.993	1.015	0.636	0.001	1.003	0.774	0.000	Down-regulated by drought in an ABA-dependent way
C0PJ51	Uncharacterized protein	0.643	0.608	0.618	0.979	0.971	0.993	0.623	0.001	0.981	0.468	0.000	Down-regulated by drought in an ABA-dependent way
C0PKD1	Uncharacterized protein	1.839	1.804	1.814	0.949	0.941	0.963	1.819	0.000	0.951	0.215	0.000	Up-regulated by drought in an ABA-dependent way
C0PL82	Uncharacterized protein	1.594	1.559	1.569	1.018	1.010	1.032	1.574	0.000	1.020	0.944	0.000	Up-regulated by drought in an ABA-dependent way
C0PLS3	Uncharacterized protein	2.105	2.070	2.080	1.088	1.080	1.102	2.085	0.000	1.090	0.126	0.000	Up-regulated by drought in an ABA-dependent way
C0PMR0	Uncharacterized protein	0.580	0.545	0.555	0.968	0.960	0.982	0.560	0.001	0.970	0.555	0.000	Down-regulated by drought in an ABA-dependent way
C0PMS5	Uncharacterized protein	0.406	0.371	0.381	1.144	1.136	1.158	0.386	0.000	1.146	0.035	0.000	Down-regulated by drought in an ABA-dependent way
C0PMV2	Uncharacterized protein	1.814	1.779	1.789	1.004	0.996	1.018	1.794	0.000	1.006	0.904	0.000	Up-regulated by drought in an ABA-dependent way
C0PN61	Uncharacterized protein	0.393	0.358	0.368	0.900	0.892	0.914	0.373	0.000	0.902	0.103	0.000	Down-regulated by drought in an ABA-dependent way
C4J1S1	Uncharacterized protein	0.675	0.640	0.650	0.916	0.908	0.930	0.655	0.002	0.918	0.154	0.000	Down-regulated by drought in an ABA-dependent way
C4J3X0	Uncharacterized protein	0.448	0.413	0.423	0.809	0.801	0.823	0.428	0.000	0.811	0.015	0.000	Down-regulated by drought in an ABA-dependent way
C4J8X4	Uncharacterized protein	0.679	0.644	0.654	1.002	0.994	1.016	0.659	0.002	1.004	0.936	0.000	Down-regulated by drought in an ABA-dependent way
C4J9M6	Uncharacterized protein	1.990	1.955	1.965	0.993	0.985	1.007	1.970	0.000	0.995	0.920	0.000	Up-regulated by drought in an ABA-dependent way
K7TI96	Uncharacterized protein	4.551	4.516	4.526	1.001	0.993	1.015	4.531	0.000	1.003	0.952	0.000	Up-regulated by drought in an ABA-dependent way
K7U170	Uncharacterized protein	0.529	0.494	0.504	0.968	0.960	0.982	0.509	0.000	0.970	0.555	0.000	Down-regulated by drought in an ABA-dependent way
K7U195	Uncharacterized protein	0.639	0.604	0.614	0.874	0.866	0.888	0.619	0.001	0.876	0.014	0.000	Down-regulated by drought in an ABA-dependent way
K7U2V0	Uncharacterized protein	1.532	1.497	1.507	0.861	0.853	0.875	1.512	0.000	0.863	0.010	0.000	Up-regulated by drought in an ABA-dependent way
K7U658	Uncharacterized protein	2.081	2.246	2.156	1.195	1.187	1.209	2.161	0.000	1.197	0.003	0.000	Up-regulated by drought in an ABA-dependent way
K7UG66	Oxygen evolving enhancer protein 3	1.713	1.678	1.688	0.860	0.852	0.874	1.693	0.000	0.862	0.010	0.000	Up-regulated by drought in an ABA-dependent way
K7UIV2	FHA transcription factor	1.879	1.844	1.854	0.904	0.896	0.918	1.859	0.001	0.906	0.114	0.000	Up-regulated by drought in an ABA-dependent way
K7UPC4	Uncharacterized protein	0.654	0.619	0.629	0.964	0.956	0.978	0.634	0.000	0.966	0.314	0.000	Down-regulated by drought in an ABA-dependent way
K7UTH3	Putative DUF1296 domain containing family protein	1.669	1.634	1.644	1.083	1.075	1.097	1.649	0.000	1.085	0.142	0.000	Up-regulated by drought in an ABA-dependent way
K7UUC8	Uncharacterized protein (Fragment)	0.671	0.636	0.646	1.129	1.121	1.143	0.651	0.002	1.131	0.048	0.000	Down-regulated by drought in an ABA-dependent way
K7UXB7	Uncharacterized protein	0.664	0.629	0.639	0.930	0.922	0.944	0.644	0.002	0.932	0.219	0.000	Down-regulated by drought in an ABA-dependent way
K7UZ21	Peroxidase	0.635	0.600	0.610	0.805	0.797	0.819	0.615	0.001	0.807	0.014	0.000	Down-regulated by drought in an ABA-dependent way
K7V2K4	Uncharacterized protein	2.104	2.109	2.099	1.150	1.142	1.164	2.104	0.000	1.152	0.031	0.000	Up-regulated by drought in an ABA-dependent way
K7V838	Uncharacterized protein	0.625	0.590	0.600	0.974	0.966	0.988	0.605	0.001	0.976	0.634	0.000	Down-regulated by drought in an ABA-dependent way
K7VXC0	Uncharacterized protein	1.989	1.954	1.964	0.973	0.965	0.987	1.969	0.000	0.975	0.620	0.000	Up-regulated by drought in an ABA-dependent way
K7W2T3	Uncharacterized protein (Fragment)	1.695	1.660	1.670	0.952	0.944	0.966	1.675	0.000	0.954	0.380	0.000	Up-regulated by drought in an ABA-dependent way
P00835	ATP synthase epsilon chain, chloroplastic	1.747	1.712	1.722	0.930	0.922	0.944	1.727	0.000	0.932	0.219	0.000	Up-regulated by drought in an ABA-dependent way
P09138	Cytochrome c oxidase subunit 3	0.478	0.443	0.453	0.939	0.931	0.953	0.458	0.000	0.941	0.274	0.000	Down-regulated by drought in an ABA-dependent way
P11647	NAD(P)H-quinone oxidoreductase chain 4, chloroplastic	0.650	0.615	0.625	0.914	0.906	0.928	0.630	0.001	0.916	0.146	0.000	Down-regulated by drought in an ABA-dependent way
P26566	50S ribosomal protein L20, chloroplastic	0.543	0.548	0.538	0.808	0.900	0.722	0.543	0.001	0.810	0.023	0.007	Down-regulated by drought in an ABA-dependent way
P49120	Histone H2B.4	0.685	0.650	0.660	0.842	0.834	0.856	0.665	0.000	0.844	0.006	0.000	Down-regulated by drought in an ABA-dependent way
P60138	Photosystem II reaction center protein L	2.448	2.413	2.423	0.685	0.677	0.699	2.428	0.000	0.687	0.000	0.000	Up-regulated by drought in an ABA-dependent way
Q2XX14	Non-specific lipid-transfer protein	2.569	2.634	2.744	1.041	1.033	1.055	2.649	0.000	1.043	0.220	0.000	Up-regulated by drought in an ABA-dependent way
Q49HD9	12-oxo-phytodienoic acid reductase	0.634	0.599	0.609	1.344	1.336	1.358	0.614	0.000	1.346	0.000	0.000	Down-regulated by drought in an ABA-dependent way
Q8W4W3	Putative gamma-glutamylcysteine synthetase	0.604	0.569	0.579	0.913	1.105	1.027	0.584	0.000	1.015	0.823	0.002	Down-regulated by drought in an ABA-dependent way
Q9FQB5	Glutathione S-transferase GST 24	1.523	1.508	1.478	1.011	1.203	0.825	1.503	0.000	1.013	0.914	0.011	Up-regulated by drought in an ABA-dependent way

a Each value represents the average of three biological replicates. The average is significant at a p < 0.01 level. A t-test value <0.05 is considered to be significant between ABA-deficient mutant vp5 and wild-type Vp5. D, drought stress.

**Table 4:** **Proteins with significant expression level changes only in *vp5* leaves under drought stress**.

**Accession**	**Description**	***Vp5*****: OS/control**			***vp5*****: OS/control**			***Vp5*****: OS/control**		***vp5*****: OS/contro**		***T*-test**	**Regulation of ABA and drought stress**
		**1**	**2**	**3**	**1**	**2**	**3**	**Average^a^**	***P*-value**	**Average^a^**	***P*-value**		
B4FCS4	Uncharacterized protein	1.083	1.079	1.105	2.250	2.264	2.266	1.089	0.202	2.260	0.000	0.000	Down-regulated by ABA
B4FUE3	Glutamate decarboxylase	1.111	1.107	1.133	1.554	1.547	1.561	1.117	0.115	1.554	0.001	0.000	Down-regulated by ABA
B4G1E6	Uncharacterized protein	1.129	1.025	1.101	1.879	1.889	1.899	1.085	0.265	1.889	0.000	0.000	Down-regulated by ABA
B4G227	Uncharacterized protein	1.104	1.100	1.126	1.634	1.637	1.650	1.110	0.132	1.640	0.000	0.000	Down-regulated by ABA
B6SGT3	Xylanase inhibitor protein 1	1.081	1.077	1.103	1.739	1.757	1.763	1.087	0.210	1.753	0.000	0.000	Down-regulated by ABA
B6TDN0	NADH-ubiquinone oxidoreductase 10.5 kDa subunit	0.974	0.970	0.996	1.577	1.576	1.598	0.980	0.749	1.584	0.001	0.000	Down-regulated by ABA
B6TNI6	DREPP4 protein	1.302	1.218	1.284	0.575	0.585	0.595	1.268	0.013	0.585	0.002	0.000	Up-regulated by ABA
B6TSK6	Oleoyl-acyl carrier protein thioesterase	0.973	0.969	0.995	1.640	1.653	1.661	0.979	0.737	1.651	0.000	0.000	Down-regulated by ABA
B6TTY1	Germin-like protein subfamily 1 member 11	0.980	0.976	1.002	0.413	0.409	0.426	0.986	0.822	0.416	0.001	0.000	Up-regulated by ABA
B6U297	Lipoxygenase	1.224	1.280	1.276	0.649	0.649	0.678	1.260	0.013	0.659	0.004	0.000	Up-regulated by ABA
C0PF28	Uncharacterized protein	1.077	1.073	1.099	0.647	0.657	0.673	1.083	0.228	0.659	0.004	0.000	Up-regulated by ABA
C4J6M5	Uncharacterized protein	0.871	0.867	0.893	1.901	1.892	1.912	0.877	0.004	1.902	0.000	0.000	Down-regulated by ABA
C4JC43	Putative VHS/GAT domain containing family protein	0.725	0.721	0.747	0.623	0.634	0.644	0.731	0.010	0.634	0.003	0.001	UP-regulated by ABA
E9NMH5	Glutamic-acid and lysine rich protein	0.979	0.975	1.001	1.555	1.569	1.577	0.985	0.810	1.567	0.001	0.000	Down-regulated by ABA
K7U8Z5	Uncharacterized protein	0.948	0.944	0.970	1.485	1.475	1.515	0.954	0.474	1.492	0.001	0.000	Down-regulated by ABA
K7V0W5	Uncharacterized protein	0.739	0.735	0.761	2.030	2.059	2.060	0.745	0.012	2.050	0.000	0.000	Down-regulated by ABA
K7WEH3	Uncharacterized protein	1.057	1.053	1.079	0.619	0.638	0.621	1.063	0.341	0.626	0.003	0.000	Up-regulated by ABA
Q9FER7	Putative legumain	0.820	0.816	0.842	1.569	1.579	1.580	0.826	0.041	1.576	0.001	0.000	Down-regulated by ABA
Q9LL87	Beta-glucosidase aggregating factor	1.123	1.049	1.005	1.584	1.577	1.590	1.059	0.430	1.584	0.001	0.000	Down-regulated by ABA
Q9ZTS9	Peroxidase J (Fragment)	0.715	0.719	0.723	0.414	0.424	0.434	0.719	0.008	0.424	0.001	0.000	Up-regulated by ABA

a Each value represents the average of three biological replicates. The average is significant at a p < 0.01 level. A t-test value <0.05 is considered to be significant between ABA-deficient mutant vp5 and wild-type Vp5. D, drought stress.

Among the 123 proteins with 1.5-fold changes of expression level only in *Vp5* (Table 3), the expression of 63 proteins was increased in *Vp5* under drought conditions, indicating that these proteins were up-regulated by drought stress in an ABA-dependent manner; the expression of 60 proteins was decreased under drought conditions, indicating that these proteins were down-regulated by drought stress in an ABA-dependent manner.

Among the 20 proteins with 1.5-fold changes of expression level only in *vp5* (Table 4), drought stress increased the expression of 13 proteins in *vp5* but only slightly affected their expression in *Vp5*, indicating that these proteins were down-regulated by ABA. By contrast, drought stress significantly decreased the expression of seven proteins in *vp5* but only slightly affected their expression in *Vp5*, indicating that these proteins were up-regulated by ABA.

### Chloroplast proteins involved in ABA signaling under drought stress

In the present study, 27 chloroplast proteins were found to be related to ABA in maize leaves under drought conditions (Table 5). Among these, three proteins related to the transcriptional process included an uncharacterized protein (B4FJH1) with nucleotide binding function, ribonucleoprotein A (B6T531), and FHA transcription factor (K7UIV2), of which the uncharacterized protein and ribonucleoprotein A were down-regulated by drought in an ABA-dependent manner while the FHA transcription factor was up-regulated by drought in an ABA-dependent manner; two proteins related to protein synthesis included eukaryotic translation initiation factor 3 subunit E (B4FDY7) and 50S ribosomal protein L20 (P26566), which were up- and down-regulated by drought in an ABA-dependent manner, respectively; nine proteins related to the photosynthesis process included uncharacterized proteins (B4FB57 and C0PLS3) with chlorophyll binding function, chlorophyll a-b binding protein 2 (B6STN4), photosystem I reaction center subunit XI (B6SXR2), an uncharacterized protein (K7U2V0) with electron transport function, oxygen evolving enhancer protein 3 (K7UG66), NAD(P)H-quinone oxidoreductase (P11647), photosystem II reaction center protein L (P60138), and ATP synthase epsilon chain (P00835), of which NAD(P)H-quinone oxidoreductase chain 4 was down-regulated by drought stress in an ABA-dependent manner while the other eight proteins were up-regulated by drought stress in an ABA-dependent manner. In addition, there were five enzymes—an uncharacterized protein (B4F9Q3) with glutathione_S-Trfase_N, 3-oxoacyl-[acyl-carrier-protein] synthase (B6TGG7), aldo-keto reductase yakc (B6TYB4), an uncharacterized protein (B8A310) with hydrolase activity, and putative gamma-glutamylcysteine synthetase (Q8W4W3)—and seven proteins with unknown function (B4FBH1, B4FDK8, B4FMW4, B4FPH3, C0HFZ5, K7WEH3, and B4FMW4).

**Table 5:** **Chloroplast proteins with significant expression level changes in *Vp5* or *vp5* leaves under drought stress**.

**Accession**	**Description**	***Vp5*****: OS/control**			***vp5*****: OS/control**			***Vp5*****: OS/control**		***vp5*****: OS/contro**		***T*-test**	**Regulation of ABA and drought stress**
		**1**	**2**	**3**	**1**	**2**	**3**	**Average^a^**	***P*-value**	**Average^a^**	***P*-value**		
B4F9Q3	Uncharacterized protein	1.825	1.890	1.850	0.916	0.898	0.910	1.855	0.000	0.908	0.188	0.000	Up-regulated by drought in an ABA-dependent way
B4FB57	Uncharacterized protein	2.096	2.001	2.131	1.031	1.023	1.045	2.076	0.000	1.033	0.600	0.000	Up-regulated by drought in an ABA-dependent way
B4FBH1	Uncharacterized protein	0.677	0.642	0.652	1.015	1.007	1.029	0.657	0.004	1.017	0.784	0.000	Down-regulated by drought in an ABA-dependent way
B4FDK8	Uncharacterized protein	1.593	1.558	1.568	1.057	1.049	1.071	1.573	0.001	1.059	0.367	0.000	Up-regulated by drought in an ABA-dependent way
B4FDY7	Eukaryotic translation initiation factor 3 subunit E	1.631	1.596	1.606	1.249	1.241	1.263	1.611	0.000	1.251	0.001	0.000	Up-regulated by drought in an ABA-dependent way
B4FJH1	Uncharacterized protein	0.500	0.465	0.475	1.024	1.016	1.038	0.480	0.001	1.026	0.678	0.000	Down-regulated by drought in an ABA-dependent way
B4FMW4	Uncharacterized protein	0.601	0.613	0.526	0.546	0.545	0.542	0.580	0.003	0.544	0.001	0.334	Down-regulated by drought in an ABA-independent way
B4FPH3	Uncharacterized protein	1.610	1.575	1.585	1.028	1.020	1.042	1.590	0.001	1.030	0.633	0.000	Up-regulated by drought in an ABA-dependent way
B6STN4	Chlorophyll a-b binding protein 2	1.641	1.606	1.616	0.861	0.853	0.875	1.621	0.000	0.863	0.008	0.000	Up-regulated by drought in an ABA-dependent way
B6SXR2	Photosystem I reaction center subunit XI	1.604	1.569	1.579	0.837	0.829	0.851	1.584	0.001	0.839	0.005	0.000	Up-regulated by drought in an ABA-dependent way
B6T531	Ribonucleoprotein A	0.618	0.583	0.593	0.748	0.740	0.762	0.598	0.001	0.750	0.004	0.000	Down-regulated by drought in an ABA-dependent way
B6TGG7	3-oxoacyl-[acyl-carrier-protein] synthase	0.624	0.589	0.599	1.355	1.347	1.369	0.604	0.001	1.357	0.002	0.000	Down-regulated by drought in an ABA-dependent way
B6TYB4	Aldo-keto reductase yakc	1.692	1.657	1.667	0.695	0.687	0.709	1.672	0.000	0.697	0.002	0.000	Up-regulated by drought in an ABA-dependent way
B8A310	Uncharacterized protein	1.898	2.063	1.973	1.096	1.088	1.110	1.978	0.000	1.098	0.142	0.000	Up-regulated by drought in an ABA-dependent way
C0HFZ5	Uncharacterized protein	1.539	1.504	1.514	0.805	0.797	0.819	1.519	0.000	0.807	0.009	0.000	Up-regulated by drought in an ABA-dependent way
C0PKD1	Uncharacterized protein	1.839	1.804	1.814	0.949	0.941	0.963	1.819	0.000	0.951	0.215	0.000	Up-regulated by drought in an ABA-dependent way
C0PLS3	Uncharacterized protein	2.105	2.070	2.080	1.088	1.080	1.102	2.085	0.000	1.090	0.126	0.000	Up-regulated by drought in an ABA-dependent way
K7U2V0	Uncharacterized protein	1.532	1.497	1.507	0.861	0.853	0.875	1.512	0.000	0.863	0.010	0.000	Up-regulated by drought in an ABA-dependent way
K7UG66	Oxygen evolving enhancer protein 3	1.713	1.678	1.688	0.860	0.852	0.874	1.693	0.000	0.862	0.010	0.000	Up-regulated by drought in an ABA-dependent way
K7UIV2	FHA transcription factor	1.879	1.844	1.854	0.904	0.896	0.918	1.859	0.001	0.906	0.114	0.000	Up-regulated by drought in an ABA-dependent way
P00835	ATP synthase epsilon chain, chloroplastic	1.747	1.712	1.722	0.930	0.922	0.944	1.727	0.000	0.932	0.219	0.000	Up-regulated by drought in an ABA-dependent way
P11647	NAD(P)H-quinone oxidoreductase chain 4, chloroplastic	0.650	0.615	0.625	0.914	0.906	0.928	0.630	0.001	0.916	0.146	0.000	Down-regulated by drought in an ABA-dependent way
P26566	50S ribosomal protein L20, chloroplastic	0.543	0.548	0.538	0.808	0.900	0.722	0.543	0.001	0.810	0.023	0.007	Down-regulated by drought in an ABA-dependent way
P60138	Photosystem II reaction center protein L	2.448	2.413	2.423	0.685	0.677	0.699	2.428	0.000	0.687	0.000	0.000	Up-regulated by drought in an ABA-dependent way
Q8W4W3	Putative gamma-glutamylcysteine synthetase	0.604	0.569	0.579	0.913	1.105	1.027	0.584	0.000	1.015	0.823	0.002	Down-regulated by drought in an ABA-dependent way
K7WEH3	Uncharacterized protein	1.057	1.053	1.079	0.619	0.638	0.621	1.063	0.341	0.626	0.003	0.000	Up-regulated by ABA
B4FMW4	Uncharacterized protein	0.601	0.613	0.526	0.546	0.545	0.542	0.580	0.003	0.544	0.001	0.334	Down-regulated by drought in an ABA-independent way

a Each value represents the average of three biological replicates. The average is significant at a p < 0.01 level. A t-test value <0.05 is considered to be significant between ABA-deficient mutant vp5 and wild-type Vp5. D, drought stress.

### ABA-regulated enzymes under drought stress

In this study, 28 enzymes were found to be implicated in drought and ABA signaling (Table 6). Six enzymes, including glutathione peroxidase (B6SU31), peroxidase (B8A1T1, K7UZ21), an uncharacterized protein (B4F9Q3) with glutathione_S-Trfase_N, glutathione S-transferase GSTU6 (B6TP77), putative gamma-glutamylcysteine synthetase (Q8W4W3), and glutathione S-transferase GST24 (Q9FQB5), were involved in glutathione synthesis and the removal of reactive oxygen species. Of the six enzymes, the expression levels of glutathione peroxidase, two peroxidases, and putative gamma-glutamylcysteine synthetase were down-regulated by drought stress in an ABA-dependent way while an uncharacterized protein, GSTU6, and GST24 were up-regulated by drought stress in an ABA-dependent way.

**Table 6:** **Enzymes with significant expression level changes in *Vp5* or *vp5* leaves under drought stress**.

**Accession**	**Description**	**V*****p5*****: OS/control**			***vp5*****: OS/control**			***Vp5*****: OS/control**		***vp5*****: OS/contro**		***T*-test**	**Regulation of ABA and drought stress**
		**1**	**2**	**3**	**1**	**2**	**3**	**Average^a^**	***P*-value**	**Average^a^**	***P*-value**		
B4F9L3	Carbonic anhydrase	0.627	0.592	0.602	0.970	0.962	0.984	0.607	0.003	0.972	0.655	0.000	Down-regulated by drought in an ABA-dependent way
B4F9Q3	Uncharacterized protein	1.825	1.890	1.850	0.916	0.898	0.910	1.855	0.000	0.908	0.188	0.000	Up-regulated by drought in an ABA-dependent way
B4FB53	Uncharacterized protein	1.527	1.492	1.502	1.060	1.052	1.074	1.507	0.001	1.062	0.346	0.000	Up-regulated by drought in an ABA-dependent way
B4FUE3	Glutamate decarboxylase	1.111	1.107	1.133	1.554	1.547	1.561	1.117	0.115	1.554	0.001	0.000	Down-regulated by ABA
B4FWT5	Soluble inorganic pyrophosphatase	1.552	1.517	1.527	0.942	0.934	0.956	1.532	0.001	0.944	0.390	0.000	Up-regulated by drought in an ABA-dependent way
B6SSB3	Anthocyanidin 5,3-O-glucosyltransferase	1.915	1.880	1.890	0.821	0.813	0.835	1.895	0.000	0.823	0.004	0.000	Up-regulated by drought in an ABA-dependent way
B6SU31	Glutathione peroxidase	0.613	0.578	0.588	0.986	0.978	1.000	0.593	0.002	0.988	0.846	0.000	Down-regulated by drought in an ABA-dependent way
B6SZK3	NAD(P)H-dependent oxidoreductase	1.857	1.822	1.832	0.933	0.925	0.947	1.837	0.000	0.935	0.326	0.000	Up-regulated by drought in an ABA-dependent way
B6SZL9	Leucine-rich repeat receptor protein kinase EXS	1.559	1.524	1.534	1.066	1.058	1.080	1.539	0.001	1.068	0.307	0.000	Up-regulated by drought in an ABA-dependent way
B6T763	Exosome complex exonuclease RRP41	0.376	0.341	0.351	1.079	1.071	1.093	0.356	0.000	1.081	0.222	0.000	Down-regulated by drought in an ABA-dependent way
B6TDN0	NADH-ubiquinone oxidoreductase 10.5 kDa subunit	0.974	0.970	0.996	1.577	1.576	1.598	0.980	0.749	1.584	0.001	0.000	Down-regulated by ABA
B6TGG7	3-oxoacyl-[acyl-carrier-protein] synthase	0.624	0.589	0.599	1.355	1.347	1.369	0.604	0.001	1.357	0.002	0.000	Down-regulated by drought in an ABA-dependent way
B6TP77	Glutathione S-transferase GSTU6	1.531	1.496	1.506	0.987	0.979	1.001	1.511	0.000	0.989	0.568	0.000	Up-regulated by drought in an ABA-dependent way
B6TSK6	Oleoyl-acyl carrier protein thioesterase	0.973	0.969	0.995	1.640	1.653	1.661	0.979	0.737	1.651	0.000	0.000	Down-regulated by ABA
B6TYB4	Aldo-keto reductase yakc	1.692	1.657	1.667	0.695	0.687	0.709	1.672	0.000	0.697	0.002	0.000	Up-regulated by drought in an ABA-dependent way
B6U297	Lipoxygenase	1.224	1.280	1.276	0.649	0.649	0.678	1.260	0.013	0.659	0.004	0.000	Up-regulated by drought in an ABA-dependent way
B6U4B9	Dual specificity protein phosphatase 4	1.605	1.620	1.680	1.059	1.051	1.073	1.635	0.000	1.061	0.376	0.000	Up-regulated by drought in an ABA-dependent way
B8A1T1	Peroxidase	0.666	0.631	0.641	1.055	1.047	1.069	0.646	0.001	1.057	0.417	0.000	Down-regulated by drought in an ABA-dependent way
C0HHB1	Carboxypeptidase	2.804	2.829	2.809	0.934	1.026	0.848	2.814	0.000	0.936	0.299	0.000	Up-regulated by drought in an ABA-dependent way
K7UZ21	Peroxidase	0.635	0.600	0.610	0.805	0.797	0.819	0.615	0.001	0.807	0.014	0.000	Down-regulated by drought in an ABA-dependent way
P00835	ATP synthase epsilon chain, chloroplastic	1.747	1.712	1.722	0.930	0.922	0.944	1.727	0.000	0.932	0.219	0.000	Up-regulated by drought in an ABA-dependent way
P09138	Cytochrome c oxidase subunit 3	0.478	0.443	0.453	0.939	0.931	0.953	0.458	0.000	0.941	0.274	0.000	Down-regulated by drought in an ABA-dependent way
P11647	NAD(P)H-quinone oxidoreductase chain 4, chloroplastic	0.650	0.615	0.625	0.914	0.906	0.928	0.630	0.001	0.916	0.146	0.000	Down-regulated by drought in an ABA-dependent way
Q49HD9	12-oxo-phytodienoic acid reductase	0.634	0.599	0.609	1.344	1.336	1.358	0.614	0.000	1.346	0.000	0.000	Down-regulated by drought in an ABA-dependent way
Q9LL87	Beta-glucosidase aggregating factor	1.123	1.049	1.005	1.584	1.577	1.590	1.059	0.430	1.584	0.001	0.000	Down-regulated by ABA
Q8W4W3	Putative gamma-glutamylcysteine synthetase	0.604	0.569	0.579	0.913	1.105	1.027	0.584	0.000	1.015	0.823	0.002	Down-regulated by drought in an ABA-dependent way
Q9FQB5	Glutathione S-transferase GST 24	1.523	1.508	1.478	1.011	1.203	0.825	1.503	0.000	1.013	0.914	0.011	Up-regulated by drought in an ABA-dependent way
Q9ZTS9	Peroxidase J (Fragment)	0.715	0.719	0.723	0.414	0.424	0.434	0.719	0.008	0.424	0.001	0.000	Down-regulated by drought, but up-regulated by ABA

a Each value represents the average of three biological replicas. The average is significant at a p < 0.01 level. A T-test value <0.05 is considered to be significant between ABA-deficient mutant vp5 and wild type Vp5. D, drought stress.

The expression levels of 12-oxo-phytodienoic acid reductase (Q49HD9, involved in jasmonic acid synthesis) and dual specificity protein phosphatase 4 (B6U4B9) were down- and up-regulated by drought stress in an ABA-dependent way, respectively; the expression levels of NAD(P)H-dependent oxidoreductase (B6SZK3) and aldo-keto reductase yakc (B6TYB4), belonging to the monomeric NADPH-dependent oxidoreductase family, were up-regulated by drought stress in an ABA-dependent manner; the expression levels of mitochondrion cytochrome c oxidase subunit 3 (P09138) and chloroplast NAD(P)H-quinone oxidoreductase chain 4 (P11647) were down-regulated by drought stress in an ABA-dependent manner, indicating that the electron transport of mitochondria and chloroplasts was inhibited under drought conditions; the expression levels of uncharacterized protein (B4FB53) belonging to the ubiquitin-conjugating enzyme family, soluble inorganic pyrophosphatase, leucine-rich repeat receptor protein kinase EXS (B6SZL9), and chloroplast ATP synthase epsilon chain (P00835) were up-regulated by drought stress in an ABA-dependent way.

**Figure 7:**
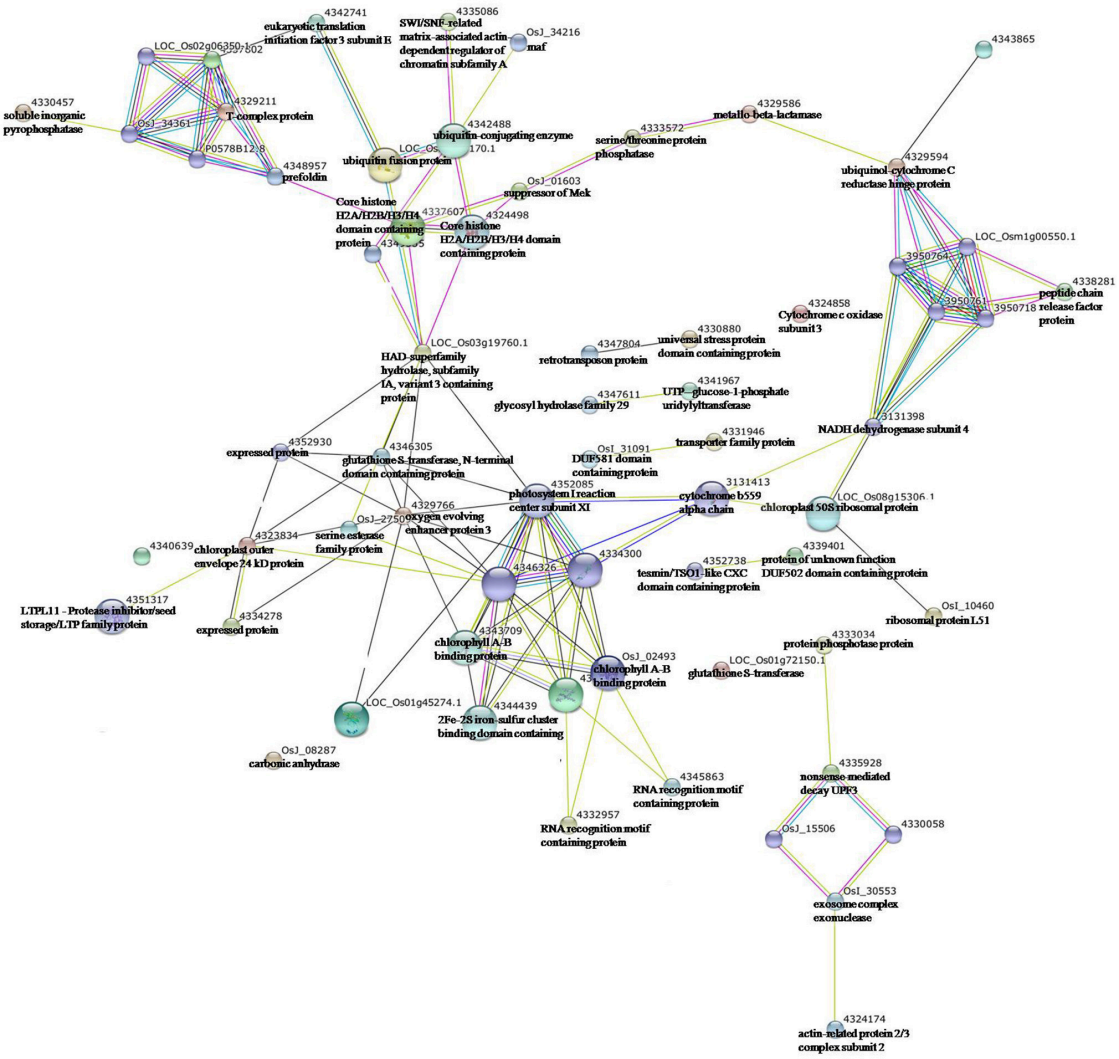
**Protein-protein interaction network analysis among the significantly expressed proteins in maize *Vp5* and *vp5* leaves under drought stress using String software**.

To uncover the interactions among these enzymes, especially protein kinases/phosphatases, with other proteins exhibiting significantly changed expression levels under drought stress, protein-protein interaction analysis was conducted using STRING software (Figure 7). In the network of interactions, five enzymes were found to play core roles in the response of maize leaves to drought stress, which included ubiquitin-conjugating enzyme (4342488), serine/threonine protein phosphatase (4333572), HAD-superfamily hydrolase (LOC_Os03g19760.1), NADH dehydrogenase subunit 4 (3131398), and glutathione S-transferase (4346305).

Ubiquitin-conjugating enzyme (4342488) had interactions with core histone H2A/H2B/H3/H4 domain containing protein (4324498), SWI/SNF-related matrix-associated actin-dependent regulator (4335086) of chromatin subfamily A, and ubiquitin fusion protein (LOC_Os03g13170.1), which further interacted with core histone H2A/H2B/H3/H4 domain containing protein, eukaryotic translation initiation factor 3 subunit E (4342741), and maf (OsJ_34216). Serine/threonine protein phosphatase (4333572) had interactions with suppressor of Mek (OsJ_01603) and metallo-beta-lactamase (4329586), while they further interacted with core histone H2A/H2B/H3/H4 domain containing protein (4324498) and ubiquinol-cytochrome C reductase hinge protein (4329594), respectively; NADH dehydrogenase subunit 4 (3131398) had interactions with cytochrome b559 alpha chain (3131413) and chloroplast 50S ribosomal protein L20 (LOC_Os08g15306.1), while they further interacted with photosystem I reaction center subunit XI (4352085) and ribosomal protein L51 (OsI_10460), respectively.

HAD-superfamily hydrolase (LOC_Os03g19760.1) had interactions with core histone H2A/H2B/H3/H4 domain containing protein (4324498), expressed protein (4352930), glutathione S-transferase (4346305), photosystem I reaction center subunit XI (4352085), and oxygen evolving enhancer protein 3 (4329766), while glutathione S-transferase also had interactions with these proteins. In addition, exosome complex exonuclease (OsI_30553) had an interaction with actin-related protein 2/3 complex subunit 2 (4324174). The results indicated that the five enzymes might have an active role in protein defense/degradation and photosynthesis protection under drought stress (maize protein query sequences matched to rice protein query sequences; see Supplementary Table S2).

### Proteins related to stimuli response under drought stress

Among 150 differentially expressed proteins, 21 proteins were characterized as “response to stimulus” (Figures 5, 6A, Table 7). Among the 21 proteins, the expression levels of three proteins (B4FBV4, B4FBY1, and C0HF37) belonging to the small heat shock protein (sHSP) family were up-regulated by drought stress in an ABA-dependent manner; two peroxidases (B8A1T1, K7UZ21) were down-regulated by drought in an ABA-dependent way, and peroxidase (Q9ZTS9) was down-regulated by ABA; DHN2-like protein (B7U627) and an uncharacterized protein (B4G1H1) of the dehydrin family, FHA transcription factor (K7UIV2) and DREPP4 protein (B6TNI6) were up-regulated by drought stress in an ABA-dependent manner. In particular, the expression of two uncharacterized proteins (C0PLS3 with ADP binding function, K7TI96 with nucleotide binding function) had 4.53- and 5.23-fold changes in *Vp5* expression, while there was no obvious difference in expression level in *vp5* under drought stress conditions.

**Table 7:** **Proteins related to stimuli response under drought stress**.

**Accession**	**Description**	***Vp5*****: OS/control**			***vp5*****: OS/control**			***Vp5*****: OS/control**		***vp5*****: OS/contro**		***T*-test**	**Regulation of ABA and drought stress**
		**1**	**2**	**3**	**1**	**2**	**3**	**Average^a^**	***P*-value**	**Average^a^**	***P*-value**		
B4FBV4	Chaperone protein dnaJ 10 isoform 1	1.705	1.793	1.740	0.934	0.926	0.948	1.746	0.000	0.936	0.332	0.000	Up-regulated by drought in an ABA-dependent way
B4FBY1	Chaperone protein dnaJ	1.646	1.611	1.621	0.956	0.928	0.930	1.626	0.000	0.938	0.349	0.000	Up-regulated by drought in an ABA-dependent way
B4FFW5	Uncharacterized protein	1.823	1.848	1.828	0.949	1.041	0.863	1.833	0.000	0.951	0.561	0.000	Up-regulated by drought in an ABA-dependent way
B4G1H1	Uncharacterized protein	1.599	1.564	1.574	1.295	1.287	1.309	1.579	0.001	1.297	0.007	0.000	Up-regulated by drought in an ABA-dependent way
B6STN4	Chlorophyll a-b binding protein 2	1.641	1.606	1.616	0.861	0.853	0.875	1.621	0.000	0.863	0.008	0.000	Up-regulated by drought in an ABA-dependent way
B6SU31	Glutathione peroxidase	0.613	0.578	0.588	0.986	0.978	1.000	0.593	0.002	0.988	0.846	0.000	Down-regulated by drought in an ABA-dependent way
B6T531	Ribonucleoprotein A	0.618	0.583	0.593	0.748	0.740	0.762	0.598	0.001	0.750	0.004	0.000	Down-regulated by drought in an ABA-dependent way
B7U627	DHN2-like protein	1.776	1.741	1.751	1.147	1.139	1.161	1.756	0.000	1.149	0.041	0.000	Up-regulated by drought in an ABA-dependent way
B8A1T1	Peroxidase	0.666	0.631	0.641	1.055	1.047	1.069	0.646	0.001	1.057	0.417	0.000	Down-regulated by drought in an ABA-dependent way
C0HF37	Uncharacterized protein	1.620	1.632	1.545	0.504	0.518	0.514	1.599	0.002	0.512	0.001	0.000	Up-regulated by drought in an ABA-dependent way
C0PLS3	Uncharacterized protein	2.105	2.070	2.080	1.088	1.080	1.102	2.085	0.000	1.090	0.126	0.000	Up-regulated by drought in an ABA-dependent way
K7TI96	Uncharacterized protein	4.551	4.516	4.526	1.001	0.993	1.015	4.531	0.000	1.003	0.952	0.000	Up-regulated by drought in an ABA-dependent way
K7TKJ3	Uncharacterized protein	5.551	5.063	5.076	0.621	0.635	0.632	5.230	0.000	0.629	0.003	0.000	Up-regulated by drought in an ABA-dependent way
K7UIV2	FHA transcription factor	1.879	1.844	1.854	0.904	0.896	0.918	1.859	0.001	0.906	0.114	0.000	Up-regulated by drought in an ABA-dependent way
K7UZ21	Peroxidase	0.635	0.600	0.610	0.805	0.797	0.819	0.615	0.001	0.807	0.014	0.000	Down-regulated by drought in an ABA-dependent way
Q8W4W3	Putative gamma-glutamylcysteine synthetase	0.604	0.569	0.579	0.913	1.105	1.027	0.584	0.000	1.015	0.823	0.002	Down-regulated by drought in an ABA-dependent way
B6TNI6	DREPP4 protein	1.302	1.218	1.284	0.575	0.585	0.595	1.268	0.013	0.585	0.002	0.000	Up-regulated by drought in an ABA-dependent way
B6U297	Lipoxygenase	1.224	1.280	1.276	0.649	0.649	0.678	1.260	0.013	0.659	0.004	0.000	Up-regulated by drought in an ABA-dependent way
E9NMH5	Glutamic-acid and lysine rich protein	0.979	0.975	1.001	1.555	1.569	1.577	0.985	0.810	1.567	0.001	0.000	Down-regulated by ABA
K7WEH3	Uncharacterized protein	1.057	1.053	1.079	0.619	0.638	0.621	1.063	0.341	0.626	0.003	0.000	Up-regulated by ABA
Q9ZTS9	Peroxidase J (Fragment)	0.715	0.719	0.723	0.414	0.424	0.434	0.719	0.008	0.424	0.001	0.000	Down-regulated by drought, but up-regulated by ABA

a Each value represents the average of three biological replicates. The average is significant at a p < 0.01 level. A t-test value <0.05 is considered to be significant between ABA-deficient mutant vp5 and wild-type Vp5. D, drought stress.

### Main signaling pathways mediated by ABA under drought stress

Based on the KEGG analysis, signaling pathways in which the differentially expressed proteins were involved for *Vp5* (Table 3) and *vp5* (Table 4) under drought stress were classified into 48 and 26 categories, respectively (for proteins corresponding to each signaling pathway, see Supplementary Tables S3, S4). For *Vp5* (Figure 4), the top three categories with the most proteins were photosynthesis (10), oxidative phosphorylation (4), glutathione metabolism (3), and RNA degradation (3). For *vp5* (Figure 5), each of 26 categories had only one protein. Particularly, 10 signal pathways were in common in *Vp5* and *vp5*; 38 were only found in *Vp5*; 16 were only found in *vp5*. Among the top 12 categories in *Vp5* (Figure 5), signaling pathways such as photosynthesis, glutathione metabolism, RNA degradation, phenylalanine metabolism, phenylpropanoid biosynthesis, ribosome, MAPK signaling pathway, and protein processing in endoplasmic reticulum were found only in *Vp5* while oxidative phosphorylation, RNA transport, alcoholism, and systemic lupus erythematosus were also found in *vp5*.

For *Vp5*, the top signaling pathway—photosynthesis—included 10 proteins (B6SP61, K7U2V0, B6SXR2, K7UG66, P00835, P60138, B6STN4, B4FB57, P11647, and C0PLS3), of which the expression of B6SP61 and P11647 was down-regulated by drought stress in an ABA-dependent manner while the expression of the other eight proteins was up-regulated by drought stress in an ABA-dependent way; the second signaling pathway—oxidative phosphorylation—included four proteins (P09138, P11647, B4FWT5, and P00835), of which P09138 and P11647 were down-regulated by drought stress in an ABA-dependent manner, and B4FWT5 and P00835 were up-regulated by drought stress in an ABA-dependent way; glutathione metabolism (including three proteins: Q8W4W3, B6SU31, and B6TP77) and RNA degradation (including three proteins: B6T763, B4FPS3, and C0PMV2) were the third top signaling pathways. Among the six proteins, the expression of Q8W4W3, B6SU31, and B6T763 was down-regulated by drought stress in an ABA-dependent manner while that of B6TP77, B4FPS3, and C0PMV2 was up-regulated by drought stress in an ABA-dependent way. These results indicated that drought stress caused major disturbances to crop photosynthesis and antioxidant defense.

## Discussion

Due to increased food demand with increasing population and crop yield loss by abnormal environmental changes, increased or stable production of crops under normal or stress conditions is necessary. Therefore, it will be helpful for food security to explore the mechanisms of crop tolerance to stress. ABA is a vital hormone and confers tolerance to abiotic stress (Sah et al., 2016). Although there are some studies concerning proteomic analysis of maize tolerance to drought stress (Benešová et al., 2012; Yang et al., 2014), there are still few studies investigating the roles of ABA in proteomic changes during maize tolerance to drought stress. In this regard, the present study focused on the roles of ABA in drought-induced proteomic changes by comparing the ABA-deficient maize mutant *vp5* and its wild-type *Vp5*.

### ABA-mediated chloroplast proteins and photosynthesis under drought stress

It has been proved that photosynthesis of C_4_ plants is highly sensitive to drought stress. With sustained drought, stomatal conductance, and CO_2_ assimilation rate decrease rapidly (for a review, see Ghannoum, 2009). Our previous results showed that ABA regulated the phosphorylation of 21 chloroplast proteins under osmotic stress conditions (Hu et al., 2015). In this study, photosynthesis was the top signaling pathway affected by drought stress. Twenty-seven chloroplast proteins were regulated by ABA in maize plants exposed to drought stress, of which 10 proteins were involved in photosynthesis. Among these 10 proteins, drought down-regulated the expression of ferredoxin-1 and NAD(P)H-quinone oxidoreductase chain 4, but up-regulated the expression of photosystem I reaction center subunit XI, oxygen evolving enhancer protein 3, ATP synthase epsilon chain, photosystem II reaction center protein L, chlorophyll a-b binding protein 2, and two uncharacterized proteins in an ABA-dependent way.

In rice, ABA and salt treatment enhanced the expression of oxygen evolving enhancer protein 2 (Abbasi and Komatsu, 2014). In *Arabidopsis*, drought stress down- and up-regulated the expression of ferredoxin-1 and ferredoxin-2 genes, respectively (Lehtimäki et al., 2010). In maize, drought enhanced the expression of ferredoxin and PsaK (photosystem I reaction center subunit) in the drought-tolerant genotype CE704 but down-regulated their expression in the drought-sensitive 2023 genotype and the expression of PsbP (23 kDa extrinsic polypeptide of photosystem II) in two genotypes (Benešová et al., 2012). These results indicate that the expression patterns of chloroplast proteins related to photosynthesis are complex under drought conditions and highly dependent on the species and genotypes of plants, and the length and severity of stress.

Of the 27 chloroplast proteins, five proteins were involved in synthesis of chloroplast proteins, including eukaryotic translation initiation factor 3 subunit E and FHA transcription factor (up-regulated by drought in an ABA-dependent way), and uncharacterized protein, ribonucleoprotein A, and 50S ribosomal protein L20 (down-regulated by drought in an ABA-dependent way). In *Arabidopsis*, over-expressing wheat eukaryotic translation initiation factor 3 subunit G enhanced the survival rate and photosynthetic efficiency of plants under drought stress (Singh et al., 2013). In maize, drought down-regulated the expression of ribonucleoprotein A in the drought-tolerant CE704 and drought-sensitive 2023 genotypes, but down-regulated the expression of ribosomal protein S18 in CE704 and up-regulated its expression in the 2023 genotype (Benešová et al., 2012). These results indicate that drought stress disturbs chloroplast protein synthesis, and eukaryotic translation initiation factor 3 could play an active role in enhancing the tolerance of crops to drought stress.

### ABA involved in drought-induced protein homeostasis and degradation

In order to gain functional activity, it is necessary for most proteins to fold into fine three-dimensional structures. However, under stress conditions, many proteins are at great risk of aberrant folding and aggregation. In order to avoid these hazards, cells need to depend on a complex network of molecular chaperones to prevent aggregation and promote efficient folding (Hartl et al., 2011; Kim et al., 2013). Recent advances showed that drought increased the expression levels of several sHSPs in maize leaves but decreased their expression in maize kernels (Benešová et al., 2012; Yang et al., 2014). Our previous study indicated that ABA regulated the changes in phosphorylation level of several sHSPs in maize leaves under drought stress (Hu et al., 2015). In the present study, drought stress enhanced the expression of three sHSPs (C0HF37, B4FBV4, and B4FBY1) but decreased the expression of HSP70 (B6SZ69) in an ABA-dependent manner. Taken together, these results show that HSPs as chaperons play extensive roles in maize endurance to drought stress.

Chaperone T-complex protein 1 has been extensively studied in humans and animals (Sinha et al., 2014; Wu et al., 2015), but the function of chaperone T-complex protein 1 remains poorly understood in plants. In this study, drought significantly elevated the expression of T-complex protein 1 subunits zeta (B6U118) and delta (C0HGT5) in an ABA-dependent way. This study therefore extends our understanding of T-complex protein 1 function in plants and is also the first research to our knowledge identifying the role of T-complex protein 1 in the maize response to drought stress.

The ubiquitin-dependent proteolytic pathway degrades most proteins and is the primary proteolysis mechanism in eukaryotic cells. Ubiquitination involves the successive action of at least three enzymes: ubiquitin-activating enzyme, ubiquitin-conjugating enzyme, and ubiquitin ligase (Zhang et al., 2015). In the current study, ubiquitin-conjugating enzyme (B4FB53) was obviously increased by drought stress in an ABA-dependent manner and had interactions with six proteins under drought conditions, including ubiquitin fusion protein (B6SHW9). In addition, drought increased the expression levels of carboxypeptidase (C0HHB1) and an uncharacterized protein (B4FEE1) with serine-type endopeptidase activity. In amaranth leaves, the content of ubiquitin conjugating enzyme was also enhanced under drought stress (Huerta-Ocampo et al., 2009). Our previous study indicated that the phosphorylation levels of eight ubiquitin proteins were significantly changed in maize leaves under drought stress (Hu et al., 2015). Taken together, these results suggest a higher rate of unnecessary or damaged protein degradation under drought conditions.

### Antioxidative proteins regulated by ABA under drought stress

Oxidative stress refers to the imbalance of reactive oxygen species removal and the antioxidant defense system, which can result in the oxidative damage of many macromolecules and an excessive reduction of electron transport chain components in chloroplasts, mitochondria, and various detoxification processes (Cruz de Carvalho, 2008). Therefore, it is necessary for plants to start fine regulation mechanisms to remove reactive oxygen species under stress conditions. In the present study, nine proteins involved in processes related to redox homeostasis were differentially regulated: three isoforms of glutathione S-transferase, glutathione peroxidase, NADH-ubiquinone oxidoreductase 10.5 kDa subunit, NAD(P)H-quinone oxidoreductase chain 4, and three isoforms of peroxidase (Table 6).

Glutathione S-transferase can reduce H_2_O_2_ to its corresponding hydroxyl compounds, which may remediate oxidative damage of membranes. Glutathione peroxidase can active with and remove H_2_O_2_. In this study, drought stress increased the expression levels of three glutathione S-transferase isoforms but decreased the expression levels of glutathione peroxidase and three peroxidase isoforms in an ABA-dependent manner. The protein-protein interaction network analysis indicated that glutathione S-transferase had interactions with six proteins, suggesting its important role in antioxidative defense. Other studies indicated that the expression levels of glutathione S-transferase and glutathione peroxidase were scarcely affected in drought-tolerant maize genotypes but were significantly increased in drought-sensitive maize genotypes under drought conditions (Benešová et al., 2012; Yang et al., 2014). Taken together, these results show that the cooperative relations among the antioxidative proteins play an important role in redox homeostasis under drought conditions.

Dehydrins and xylanase inhibitor proteins act as cell rescue/defense-related proteins. Dehydrins play important roles in protecting the stability of membrane proteins, adjusting cell osmotic pressure, and stabilizing and preventing the denaturation of macromolecules (Close, 1996; Mohammadkhani and Heidari, 2008). Xylanase inhibitor proteins are believed to play a role in plant defensive reactions (Dornez et al., 2010; Vasconcelos et al., 2011). A previous study indicated that the expression levels of dehydrin and xylanase inhibitor protein 1 were barely affected in the drought-tolerant maize line Lo964 but were significantly increased in the drought-sensitive line B73 under drought conditions (Yang et al., 2014). In the maize drought-tolerant CE704 and drought-sensitive 2023 genotypes, the expression of dehydrin RAB-17 was increased under drought stress, but the increase was greater in CE704 than in the 2023 genotype. However, drought stress increased the expression of xylanase inhibitor (TAXI-IV) in the drought-tolerant CE704 genotype but decreased its expression in the drought-sensitive 2023 genotype (Benešová et al., 2012). In the current study, two isoforms of dehydrin were up-regulated by drought in an ABA-dependent manner; xylanase inhibitor protein 1 was up-regulated by drought but down-regulated by ABA. These results indicate that the regulation of drought stress by dehydrin and xylanase inhibitor protein depends on the plant species, genotype, and the severity/length of drought stress.

Overall, the application of the maize ABA-deficient mutant *vp5* and its wild-type *Vp5* was very useful to identify drought-response proteins involved in ABA signaling pathways. Among the 150 proteins identified, 67 proteins were up-regulated by drought in an ABA-dependent way; 60 proteins were down-regulated by drought in an ABA-dependent way. Under drought stress, the top three signaling pathways affected were photosynthesis, oxidative phosphorylation (mainly chloroplast-mediated ATP synthesis), and glutathione metabolism, indicating that ABA plays an important role in regulating photosynthesis, ATP synthesis, and antioxidative reactions in maize under drought stress. In addition, stimuli-response proteins (such as chaperone proteins and dehydrins), antioxidative proteins (such as glutathione S-transferase), and proteins for protein degradation (such as ubiquitin-conjugating enzyme, carboxypeptidase and endopeptidase) might have more effective roles in maize tolerance to drought stress. The present study provides a basis for further understanding the mechanisms and roles of ABA in maize responses to drought stress.

## Author contributions

XH conceived the study and participated in its design. YZ and YW carried out the experiments. JW contributed samples. WW revised the manuscript. XH and HY analyzed the data and drafted the manuscript.

### Conflict of interest statement

The authors declare that the research was conducted in the absence of any commercial or financial relationships that could be construed as a potential conflict of interest.

## Supplementary material

Supplementary Table S1 **Primers used in this study**.

Supplementary Table S2 **Maize proteins corresponding to rice proteins in the network of protein interaction in maize *Vp5* seedlings under drought stress**.

Supplementary Table S3 **Map name of pathways induced by drought stress in maize *Vp5***.

Supplementary Table S4 **Map name of pathways induced by drought stress in maize *vp5***.

Supplementary Excel S1 **Indentified peptides for the first independent biological experiment**.

Supplementary Excel S2 **Indentified peptides for the second independent biological experiment**.

Supplementary Excel S3 **Indentified peptides for the third independent biological experiment**.
