# Supplementary material for: Quantitative Proteomic Analyses Identify ABA-Related Proteins and Signal Pathways in Maize Leaves under Drought Conditions
Source: Front Plant Sci. 2016 Dec 8;7:1827. doi: 10.3389/fpls.2016.01827 (PMC5143342; doi:10.3389/fpls.2016.01827)
Supplement: Supplementary Table S1 — Primers used in this study. [file Table1.DOC]

**Supplemental Table 1. Primers used in this study**

| **Target** | **Protein Accession** | **Gene ID** | **Primer name** | **Primer sequences (5’ to 3’)** |
| --- | --- | --- | --- | --- |
| Chlorophyll a-b binding protein 2 | B6STN4 | NM_001154167.1 | Chlorophyll a-b binding protein 2-F | AGAGCATCCTCGCCATCT |
|  |  |  | Chlorophyll a-b binding protein 2-R | AGAGCATCCTCGCCATCT |
| Chloroplast outer envelope 24 kD protein | B6TM56 | XM_008676257.1 | Chloroplast outer envelope 24 kD protein-F | CGTCTACGGTGCGTTTCTT |
|  |  |  | Chloroplast outer envelope 24 kD protein-R | GGTGAGTCCTTTCTGTCCTG |
| Glutathione S-transferase GST 24 | Q9FQB5 | NM_001111518.1 | Glutathione S-transferase GST 24-F | GCTTCTGGGCTGACTTCA |
|  |  |  | Glutathione S-transferase GST 24-R | TCACGACCTCATCGGAGA |
| Glutathione S-transferase GSTU6 | B6TP77 | NM_001111528.1 | Glutathione S-transferase GSTU6-F | TGATGCTGTTCGGGTCGT |
|  |  |  | Glutathione S-transferase GSTU6-R | ACTGGAGGATAATGACGG |
| Heat shock cognate 70 kDa protein 2 | B6SZ69 | NM_001174225.1 | Heat shock cognate 70 kDa protein 2-F | CAAGAACGCTGTCGTCAC |
|  |  |  | Heat shock cognate 70 kDa protein 2-R | ACTCCTGGACGAAGTGGT |
| NAD(P)H-dependent oxidoreductase | B6SZK3 | NM_001154774.2 | NAD(P)H-dependent oxidoreductase-F | CGTGGACCTGTTCCTGAT |
|  |  |  | NAD(P)H-dependent oxidoreductase-R | GGCGATTTGTTTGAGGAC |
| Photosystem II reaction center protein L | P60138 | NC_001666.2 | Photosystem II reaction center protein L-F | ATGACACAATCAAACCCGAA |
|  |  |  | Photosystem II reaction center protein L-R | TCAATTGAAG AAGTAATTGG |
| Putative RING zinc finger domain superfamily protein | B4F9U4 | NM_001154069.1 | Putative RING zinc finger domain superfamily protein-F | TTGGAACCTCGCCTTCAT |
|  |  |  | Putative RING zinc finger domain superfamily protein-R | CGCATCACGGGTAAGAAC |
| actin1 | B6T5K6 | NM_001155179.1 | actin1-F | AAATGACGCAGATTATGTTTGA |
|  |  |  | actin1-R | GCTCGTAGTGAGGGAGTACC |
