# Supplementary material for: Quantitative Proteomic Analyses Identify ABA-Related Proteins and Signal Pathways in Maize Leaves under Drought Conditions
Source: Front Plant Sci. 2016 Dec 8;7:1827. doi: 10.3389/fpls.2016.01827 (PMC5143342; doi:10.3389/fpls.2016.01827)
Supplement: Supplementary Table S2 — Maize proteins corresponding to rice proteins in the network of protein interaction in maize Vp5 seedlings under drought stress. [file Table2.DOC]

**Table S2︱Maize proteins corresponding to rice proteins in the network of protein interaction in maize *Vp*5 seedlings under drought stress**

| Maize query sequence | Rice query sequence | STRING protein | Identity | Bitscore |
| --- | --- | --- | --- | --- |
| P00835 | 3131461 | ATP synthase CF1 epsilon chain | 98% | 270 |
| P09138 | 3950764 | Cytochrome c oxidase subunit 3 | 98% | 517 |
| P11647 | 3131398 | NADH dehydrogenase subunit 4 | 96% | 924 |
| P26566 | LOC_Os08g15306.1 | chloroplast 50S ribosomal protein L20 | 89% | 212 |
| P49120 | 4324498 | Core histone H2A/H2B/H3/H4 domain containing protein | 86% | 196 |
| P60138 | LOC_Os06g39734.1 | hypothetical protein | 100% | 77 |
| B4F9L3 | LOC_Os01g45274.1 | carbonic anhydrase, chloroplast precursor | 81% | 338 |
| B4F9Q3 | 4346305 | glutathione S-transferase, N-terminal domain containing protein | 82% | 208 |
| B4F9U4 | 4330296 | zinc finger family protein | 82% | 575 |
| B4FA94 | OsI_10460 | ribosomal protein L51 | 97% | 239 |
| B4FB53 | 4342488 | ubiquitin-conjugating enzyme | 97% | 311 |
| B4FB57 | 4343709 | chlorophyll A-B binding protein | 90% | 166 |
| B4FB96 | 4330251 | harpin-induced protein 1 domain containing protein | 60% | 200 |
| B4FBH1 | 4336884 | DAG protein, chloroplast precursor | 93% | 375 |
| B4FBV4 | 4339491 | chaperone protein dnaJ 10 | 85% | 593 |
| B4FBY1 | OsI_29447 | heat shock protein DnaJ | 73% | 175 |
| B4FDK8 | 4343865 | expressed protein | 80% | 363 |
| B4FDY7 | 4342741 | eukaryotic translation initiation factor 3 subunit E | 87% | 804 |
| B4FEE1 | 4328983 | OsSub18 - Putative Subtilisin homologue | 58% | 399 |
| B4FFW5 | 4330880 | universal stress protein domain containing protein | 89% | 306 |
| B4FI24 | OsI_31091 | DUF581 domain containing protein | 66% | 187 |
| B4FJ71 | 4347804 | retrotransposon protein | 86% | 219 |
| B4FJH1 | 4345863 | RNA recognition motif containing protein | 76% | 258 |
| B4FK45 | 4352930 | expressed protein | 67% | 413 |
| B4FMW4 | OsJ_14164 | DUF647 domain containing protein | 86% | 769 |
| B4FP86 | 4342181 | expressed protein | 77% | 409 |
| B4FPH3 | 4331827 | ataxin-2 C-terminal region family protein | 62% | 625 |
| B4FPM5 | OsJ_29831 | jmjC domain-containing protein 5 | 83% | 564 |
| B4FPS3 | 4325594 | expressed protein | 87% | 357 |
| B4FQN6 | 4331946 | transporter family protein | 81% | 867 |
| B4FRG9 | 4334367 | fasciclin domain containing protein | 88% | 311 |
| B4FWT5 | 4330457 | soluble inorganic pyrophosphatase | 88% | 369 |
| B4FX06 | 4352197 | amidohydrolase | 80% | 743 |
| B4FX77 | OsJ_22393 | zinc finger C-x8-C-x5-C-x3-H type family protein | 63% | 407 |
| B4FZ22 | 4330748 | endonuclease/exonuclease/phosphatase family domain containing protein | 77% | 891 |
| B4G1A3 | 4334183 | cytochrome P450 | 72% | 734 |
| B4G1H1 | 4330265 | dehydrin | 57% | 190 |
| B6SHW9 | LOC_Os03g13170.1 | ubiquitin fusion protein | 99% | 263 |
| B6SJL2 | 4352567 | thaumatin family domain containing protein | 74% | 293 |
| B6SK13 | 4330515 | phosphoribosyl transferase | 92% | 588 |
| B6SMB0 | 4329594 | ubiquinol-cytochrome C reductase hinge protein | 87% | 132 |
| |B6SP61 | 4344439 | 2Fe-2S iron-sulfur cluster binding domain containing protein | 63% | 183 |
| B6SSB3 | 4343321 | UDP-glucoronosyl and UDP-glucosyl transferase domain containing protein | 60% | 528 |
| B6STN4 | 4324599 | chlorophyll A-B binding protein | 91% | 482 |
| B6SU20 | 4337480 | OsFBT6 - F-box and tubby domain containing protein | 73% | 677 |
| B6SU31 | 4330235 | glutathione peroxidase | 91% | 310 |
| B6SV53 | OsJ_14755 | ctr copper transporter family protein | 56% | 123 |
| B6SXR2 | 4352085 | photosystem I reaction center subunit XI, chloroplast precursor | 92% | 332 |
| B6SZ69 | 4332413 | DnaK family protein | 94% | 1266 |
| B6SZK3 | 4347980 | oxidoreductase, aldo/keto reductase family protein | 67% | 469 |
| B6SZL9 | 4336539 | protein kinase domain containing protein | 74% | 513 |
| B6T022 | 4334617 | BBTI13 - Bowman-Birk type bran trypsin inhibitor precursor | 56% | 78.2 |
| B6T072 | 4335046 | hypothetical protein | 87% | 124 |
| B6T1H8 | 4340639 | B12D protein | 91% | 136 |
| B6T1Q2 | 4350379 | 4F5 protein family protein | 85% | 78.2 |
| B6T3J3 | 4337607 | Core histone H2A/H2B/H3/H4 domain containing protein | 81% | 231 |
| B6T531 | 4332957 | RNA recognition motif containing protein | 73% | 350 |
| B6T763 | OsI_30553 | exosome complex exonuclease | 93% | 468 |
| B6T8S7 | 4349533 | FGFR1 oncogene partner | 89% | 453 |
| B6TGG7 | 4340392 | 3-oxoacyl-synthase | 91% | 839 |
| B6TGS2 | 4333937 | fb27 | 84% | 301 |
| B6TLU5 | 4324858 | LTPL101 - Protease inhibitor/seed storage/LTP family protein precursor | 81% | 183 |
| B6TM56 | 4323834 | chloroplast outer envelope 24 kD protein | 68% | 142 |
| B6TP77 | 4338787 | glutathione S-transferase | 82% | 387 |
| B6TRQ5 | 4352738 | tesmin/TSO1-like CXC domain containing protein | 75% | 456 |
| B6TUT9 | 4333108 | expressed protein | 51% | 131 |
| B6TVU2 | 4348957 | prefoldin | 89% | 271 |
| B6TWU1 | 4324590 | expressed protein | 82% | 203 |
| B6TYB4 | 4342382 | oxidoreductase, aldo/keto reductase family protein | 85% | 617 |
| B6TYM4 | 4343742 | C2 domain containing protein | 74% | 584 |
| B6U118 | 4337802 | T-complex protein | 97% | 1064 |
| B6U4B9 | 4330549 | dual specificity protein phosphatase | 70% | 388 |
| B6U5V4 | 4340665 | caleosin related protein | 82% | 317 |
| B6U7X1 | OsJ_34216 | maf | 91% | 384 |
| B6UAH3 | 4324890 | expressed protein | 82% | 137 |
| B6UEB0 | 4337884 | LTPL104 - Protease inhibitor/seed storage/LTP family protein precursor | 93% | 152 |
| B6UG46 | 4339705 | pex14 | 65% | 194 |
| B7U627 | 4330265 | dehydrin | 58% | 184 |
| B8A1T1 | 4332175 | peroxidase precursor | 77% | 493 |
| B8A310 | LOC_Os03g19760.1 | HAD-superfamily hydrolase, subfamily IA, variant 3 containing protein | 68% | 481 |
| C0HEI0 | 4331519 | CHIT16 - Chitinase family protein precursor | 76% | 395 |
| C0HF37 | 4340572 | CS domain containing protein | 75% | 438 |
| C0HFZ5 | 4339073 | outer membrane protein, OMP85 family protein | 78% | 744 |
| C0HG70 | 4333572 | serine/threonine protein phosphatase | 96% | 315 |
| C0HGT5 | 4329211 | T-complex protein | 95% | 1003 |
| C0HHB1 | 4344152 | OsSCP40 - Putative Serine Carboxypeptidase homologue | 76% | 758 |
| C0HJ24 | 4329586 | metallo-beta-lactamase | 71% | 196 |
| C0P2V2 | 4333034 | protein phosphotase protein | 90% | 422 |
| C0P2V7 | 4333169 | expressed protein | 64% | 95.1 |
| C0P3Y3 | 4331683 | YT521-B-like family domain containing protein | 82% | 1077 |
| C0P5M7 | OsJ_29854 | dehydrogenase | 49% | 299 |
| C0P727 | 4341967 | UTP--glucose-1-phosphate uridylyltransferase | 87% | 1102 |
| C0PBJ1 | OsJ_08287 | caleosin related protein | 58% | 243 |
| C0PF36 | OsJ_27509 | serine esterase family protein | 82% | 389 |
| C0PFW9 | 4324174 | actin-related protein 2/3 complex subunit 2 | 92% | 533 |
| C0PIW4 | 4327179 | protein kinase domain containing protein | 78% | 290 |
| C0PJ51 | 4335086 | SWI/SNF-related matrix-associated actin-dependent regulator of chromatin subfamily A | 83% | 824 |
| C0PKD1 | 4338540 | mitochondrial carrier protein | 89% | 350 |
| C0PL82 | 4334278 | expressed protein | 68% | 228 |
| C0PLS3 | OsJ_02493 | chlorophyll A-B binding protein | 97% | 312 |
| C0PMR0 | 4326027 | respiratory burst oxidase | 92% | 577 |
| C0PMS5 | 4329170 | prefoldin | 84% | 232 |
| C0PMV2 | 4349535 | expressed protein | 53% | 280 |
| C0PN61 | 4347611 | glycosyl hydrolase family 29 | 82% | 458 |
| C4J1S1 | 4339297 | glucan endo-1,3-beta-glucosidase-like protein 3 precursor | 52% | 129 |
| C4J3X0 | 4334132 | anther-specific proline-rich protein APG precursor | 77% | 117 |
| C4J8X4 | OsJ_01603 | suppressor of Mek | 54% | 135 |
| C4J9M6 | 4348938 | expressed protein | 78% | 633 |
| K7TI96 | 4350811 | stripe rust resistance protein Yr10 | 40% | 618 |
| K7TKJ3 | 4335928 | nonsense-mediated decay UPF3 | 75% | 742 |
| K7U170 | 4338281 | peptide chain release factor protein | 90% | 541 |
| K7U195 | OsI_18395 | expressed protein | 61% | 204 |
| K7U2V0 | 3131413 | cytochrome b559 alpha chain | 98% | 164 |
| K7U658 | 4344889 | expressed protein | 30% | 72.8 |
| K7UG66 | 4329766 | oxygen evolving enhancer protein 3, identical | 77% | 236 |
| K7UIV2 | 4335984 | zeaxanthin epoxidase, chloroplast precursor | 83% | 445 |
| K7UTH3 | 4339638 | protein of unknown function DUF1296 domain containing protein | 64% | 915 |
| K7UUC8 | 4324055 | PHLOEM 2-LIKE A5 | 49% | 207 |
| K7UXB7 | 4339592 | tRNA-binding arm | 71% | 558 |
| K7UZ21 | 4339222 | peroxidase precursor | 82% | 486 |
| K7V2K | 4337554 | methionine S-methyltransferase | 49% | 80.1 |
| K7V838 | 4328079 | oxidoreductase, short chain dehydrogenase/reductase family domain containing protein | 66% | 481 |
| K7VXC0 | 4339401 | protein of unknown function DUF502 domain containing protein | 76% | 71.6 |
| K7W2T3 | OsI_04625 | expressed protein | 77% | 399 |
| |Q2XX14 | 4351317 | LTPL11 - Protease inhibitor/seed storage/LTP family protein precursor | 80% | 149 |
| Q49HD9 | 4340489 | 12-oxophytodienoate reductase | 67% | 508 |
| Q8W4W3 | GSH1-2 | glutamate--cysteine ligase, chloroplast precursor | 93% | 859 |
| Q9FQB5 | LOC_Os01g72150.1 | glutathione S-transferase | 65% | 294 |
