# Supplementary material for: Quantitative Proteomic Analyses Identify ABA-Related Proteins and Signal Pathways in Maize Leaves under Drought Conditions
Source: Front Plant Sci. 2016 Dec 8;7:1827. doi: 10.3389/fpls.2016.01827 (PMC5143342; doi:10.3389/fpls.2016.01827)
Supplement: Supplementary Table S3 — Map name of pathways induced by drought stress in maize Vp5. [file Table3.DOC]

**Table S3 | Map name of pathways induced by drought stress in maize *Vp*5**

| **Map ID** | **Map Name** | **Seqs** | **#Seqs** | **URL** |
| --- | --- | --- | --- | --- |
| ko00195 | Photosynthesis | B6SP61 K7U2V0 B6SXR2 K7UG66 P00835 P60138 B6STN4 B4FB57 P11647 C0PLS3 | 9 | <http://www.kegg.jp/kegg-bin/show_pathway?ko00195+K02639+K02707+K->  02699+K08901+K02114+K02713 |
| ko00190 | Oxidative phosphorylation | P09138 P11647 B4FWT5 P00835 | 4 | http://www.kegg.jp/kegg-bin/show_pathway?ko00190+K02262+K05575+K01507+K02114 |
| ko00480 | Glutathione metabolism | Q8W4W3 B6SU31 B6TP77 | 3 | http://www.kegg.jp/kegg-bin/show_pathway?ko00480+K01919+K00432+K00799 |
| ko03018 | RNA degradation | B6T763 B4FPS3 C0PMV2 | 3 | http://www.kegg.jp/kegg-bin/show_pathway?ko03018+K11600+K12617+K10643 |
| ko00360 | Phenylalanine metabolism | K7UZ21 B8A1T1 | 2 | http://www.kegg.jp/kegg-bin/show_pathway?ko00360+K00430+K00430 |
| ko00940 | Phenylpropanoid biosynthesis | K7UZ21 B8A1T1 | 2 | http://www.kegg.jp/kegg-bin/show_pathway?ko00940+K00430+K00430 |
| ko03010 | Ribosome | P26566 B6SHW9 | 2 | http://www.kegg.jp/kegg-bin/show_pathway?ko03010+K02887+K02927 |
| ko03013 | RNA transport | B4FDY7 K7TKJ3 | 2 | http://www.kegg.jp/kegg-bin/show_pathway?ko03013+K03250+K14328 |
| ko04010 | MAPK signaling pathway | B6SZ69 B6U4B9 | 2 | http://www.kegg.jp/kegg-bin/show_pathway?ko04010+K03283+K04459 |
| ko04141 | Protein processing in endoplasmic reticulum | C0PMS5 B6SZ69 | 2 | http://www.kegg.jp/kegg-bin/show_pathway?ko04141+K14009+K03283 |
| ko05034 | Alcoholism | P49120 B6T3J3 | 2 | http://www.kegg.jp/kegg-bin/show_pathway?ko05034+K11252+K11251 |
| ko05322 | Systemic lupus erythematosus | P49120 B6T3J3 | 2 | http://www.kegg.jp/kegg-bin/show_pathway?ko05322+K11252+K11251 |
| ko00040 | Pentose and glucuronate interconversions | C0P727 | 1 | http://www.kegg.jp/kegg-bin/show_pathway?ko00040+K12447 |
| ko00053 | Ascorbate and aldarate metabolism | C0P727 | 1 | http://www.kegg.jp/kegg-bin/show_pathway?ko00053+K12447 |
| ko00061 | Fatty acid biosynthesis | B6TGG7 | 1 | http://www.kegg.jp/kegg-bin/show_pathway?ko00061+K09458 |
| ko00363 | Bisphenol degradation | B4G1A3 | 1 | http://www.kegg.jp/kegg-bin/show_pathway?ko00363+K00517 |
| ko00450 | Selenocompound metabolism | K7V2K4 | 1 | http://www.kegg.jp/kegg-bin/show_pathway?ko00450+K08247 |
| ko00511 | Other glycan degradation | C0PN61 | 1 | http://www.kegg.jp/kegg-bin/show_pathway?ko00511+K01206 |
| ko00520 | Amino sugar and nucleotide sugar metabolism | C0P727 | 1 | http://www.kegg.jp/kegg-bin/show_pathway?ko00520+K12447 |
| ko00590 | Arachidonic acid metabolism | B6SU31 | 1 | http://www.kegg.jp/kegg-bin/show_pathway?ko00590+K00432 |
| ko00592 | alpha-Linolenic acid metabolism | Q49HD9 | 1 | http://www.kegg.jp/kegg-bin/show_pathway?ko00592+K05894 |
| ko00624 | Polycyclic aromatic hydrocarbon degradation | B4G1A3 | 1 | http://www.kegg.jp/kegg-bin/show_pathway?ko00624+K00517 |
| ko00627 | Aminobenzoate degradation | B4G1A3 | 1 | http://www.kegg.jp/kegg-bin/show_pathway?ko00627+K00517 |
| ko00780 | Biotin metabolism | B6TGG7 | 1 | http://www.kegg.jp/kegg-bin/show_pathway?ko00780+K09458 |
| ko00903 | Limonene and pinene degradation | B4G1A3 | 1 | http://www.kegg.jp/kegg-bin/show_pathway?ko00903+K00517 |
| ko00906 | Carotenoid biosynthesis | K7UIV2 | 1 | http://www.kegg.jp/kegg-bin/show_pathway?ko00906+K09838 |
| ko00910 | Nitrogen metabolism | B4F9L3 | 1 | http://www.kegg.jp/kegg-bin/show_pathway?ko00910+K01673 |
| ko00945 | Stilbenoid, diarylheptanoid and gingerol biosynthesis | B4G1A3 | 1 | http://www.kegg.jp/kegg-bin/show_pathway?ko00945+K00517 |
| ko00980 | Metabolism of xenobiotics by cytochrome P450 | B6TP77 | 1 | http://www.kegg.jp/kegg-bin/show_pathway?ko00980+K00799 |
| ko00982 | Drug metabolism - cytochrome P450 | B6TP77 | 1 | http://www.kegg.jp/kegg-bin/show_pathway?ko00982+K00799 |
| ko01212 | Fatty acid metabolism | B6TGG7 | 1 | http://www.kegg.jp/kegg-bin/show_pathway?ko01212+K09458 |
| ko03015 | mRNA surveillance pathway | K7TKJ3 | 1 | http://www.kegg.jp/kegg-bin/show_pathway?ko03015+K14328 |
| ko03040 | Spliceosome | B6SZ69 | 1 | http://www.kegg.jp/kegg-bin/show_pathway?ko03040+K03283 |
| ko04120 | Ubiquitin mediated proteolysis | B4FB53 | 1 | http://www.kegg.jp/kegg-bin/show_pathway?ko04120+K10573 |
| ko04144 | Endocytosis | B6SZ69 | 1 | http://www.kegg.jp/kegg-bin/show_pathway?ko04144+K03283 |
| ko04260 | Cardiac muscle contraction | P09138 | 1 | http://www.kegg.jp/kegg-bin/show_pathway?ko04260+K02262 |
| ko04612 | Antigen processing and presentation | B6SZ69 | 1 | http://www.kegg.jp/kegg-bin/show_pathway?ko04612+K03283 |
| ko04626 | Plant-pathogen interaction | C0PMR0 | 1 | http://www.kegg.jp/kegg-bin/show_pathway?ko04626+K13447 |
| ko04666 | Fc gamma R-mediated phagocytosis | C0PFW9 | 1 | http://www.kegg.jp/kegg-bin/show_pathway?ko04666+K05758 |
| ko04810 | Regulation of actin cytoskeleton | C0PFW9 | 1 | http://www.kegg.jp/kegg-bin/show_pathway?ko04810+K05758 |
| ko04915 | Estrogen signaling pathway | B6SZ69 | 1 | http://www.kegg.jp/kegg-bin/show_pathway?ko04915+K03283 |
| ko04918 | Thyroid hormone synthesis | B6SU31 | 1 | http://www.kegg.jp/kegg-bin/show_pathway?ko04918+K00432 |
| ko04932 | Non-alcoholic fatty liver disease (NAFLD) | P09138 | 1 | http://www.kegg.jp/kegg-bin/show_pathway?ko04932+K02262 |
| ko05010 | Alzheimer's disease | P09138 | 1 | http://www.kegg.jp/kegg-bin/show_pathway?ko05010+K02262 |
| ko05012 | Parkinson's disease | P09138 | 1 | http://www.kegg.jp/kegg-bin/show_pathway?ko05012+K02262 |
| ko05016 | Huntington's disease | P09138 | 1 | http://www.kegg.jp/kegg-bin/show_pathway?ko05016+K02262 |
| ko05100 | Bacterial invasion of epithelial cells | C0PFW9 | 1 | http://www.kegg.jp/kegg-bin/show_pathway?ko05100+K05758 |
| ko05130 | Pathogenic Escherichia coli infection | C0PFW9 | 1 | http://www.kegg.jp/kegg-bin/show_pathway?ko05130+K05758 |
| ko05131 | Shigellosis | C0PFW9 | 1 | http://www.kegg.jp/kegg-bin/show_pathway?ko05131+K05758 |
| ko05132 | Salmonella infection | C0PFW9 | 1 | http://www.kegg.jp/kegg-bin/show_pathway?ko05132+K05758 |
| ko05134 | Legionellosis | B6SZ69 | 1 | http://www.kegg.jp/kegg-bin/show_pathway?ko05134+K03283 |
| ko05145 | Toxoplasmosis | B6SZ69 | 1 | http://www.kegg.jp/kegg-bin/show_pathway?ko05145+K03283 |
| ko05160 | Hepatitis C | B4FDY7 | 1 | http://www.kegg.jp/kegg-bin/show_pathway?ko05160+K03250 |
| ko05162 | Measles | B6SZ69 | 1 | http://www.kegg.jp/kegg-bin/show_pathway?ko05162+K03283 |
| ko05164 | Influenza A | B6SZ69 | 1 | http://www.kegg.jp/kegg-bin/show_pathway?ko05164+K03283 |
| ko05169 | Epstein-Barr virus infection | B6SZ69 | 1 | http://www.kegg.jp/kegg-bin/show_pathway?ko05169+K03283 |
| ko05203 | Viral carcinogenesis | P49120 | 1 | http://www.kegg.jp/kegg-bin/show_pathway?ko05203+K11252 |
| ko05204 | Chemical carcinogenesis | B6TP77 | 1 | http://www.kegg.jp/kegg-bin/show_pathway?ko05204+K00799 |
