# Supplementary material for: Quantitative Proteomic Analyses Identify ABA-Related Proteins and Signal Pathways in Maize Leaves under Drought Conditions
Source: Front Plant Sci. 2016 Dec 8;7:1827. doi: 10.3389/fpls.2016.01827 (PMC5143342; doi:10.3389/fpls.2016.01827)
Supplement: Supplementary Table S4 — Map name of pathways induced by drought stress in maize vp5. [file Table4.DOC]

**Table S4 | Map name of pathways induced by drought stress in maize *vp*5**

| **Map ID** | **Map Name** | **Seqs** | **#Seqs** | **URL** |
| --- | --- | --- | --- | --- |
| ko00020 | Citrate cycle (TCA cycle) | C4J6M5 | 1 | http://www.kegg.jp/kegg-bin/show_pathway?ko00020+K01681 |
| ko00061 | Fatty acid biosynthesis | B6TSK6 | 1 | http://www.kegg.jp/kegg-bin/show_pathway?ko00061+K10782 |
| ko00190 | Oxidative phosphorylation | B6TDN0 | 1 | http://www.kegg.jp/kegg-bin/show_pathway?ko00190+K03946 |
| ko00250 | Alanine, aspartate and glutamate metabolism | B4FUE3 | 1 | http://www.kegg.jp/kegg-bin/show_pathway?ko00250+K01580 |
| ko00410 | beta-Alanine metabolism | B4FUE3 | 1 | http://www.kegg.jp/kegg-bin/show_pathway?ko00410+K01580 |
| ko00430 | Taurine and hypotaurine metabolism | B4FUE3 | 1 | http://www.kegg.jp/kegg-bin/show_pathway?ko00430+K01580 |
| ko00591 | Linoleic acid metabolism | B6U297 | 1 | http://www.kegg.jp/kegg-bin/show_pathway?ko00591+K15718 |
| ko00630 | Glyoxylate and dicarboxylate metabolism | C4J6M5 | 1 | http://www.kegg.jp/kegg-bin/show_pathway?ko00630+K01681 |
| ko00650 | Butanoate metabolism | B4FUE3 | 1 | http://www.kegg.jp/kegg-bin/show_pathway?ko00650+K01580 |
| ko00720 | Carbon fixation pathways in prokaryotes | C4J6M5 | 1 | http://www.kegg.jp/kegg-bin/show_pathway?ko00720+K01681 |
| ko01200 | Carbon metabolism | C4J6M5 | 1 | http://www.kegg.jp/kegg-bin/show_pathway?ko01200+K01681 |
| ko01210 | 2-Oxocarboxylic acid metabolism | C4J6M5 | 1 | http://www.kegg.jp/kegg-bin/show_pathway?ko01210+K01681 |
| ko01230 | Biosynthesis of amino acids | C4J6M5 | 1 | http://www.kegg.jp/kegg-bin/show_pathway?ko01230+K01681 |
| ko04142 | Lysosome | Q9FER7 | 1 | http://www.kegg.jp/kegg-bin/show_pathway?ko04142+K01369 |
| ko04612 | Antigen processing and presentation | Q9FER7 | 1 | http://www.kegg.jp/kegg-bin/show_pathway?ko04612+K01369 |
| ko04727 | GABAergic synapse | B4FUE3 | 1 | http://www.kegg.jp/kegg-bin/show_pathway?ko04727+K01580 |
| ko04932 | Non-alcoholic fatty liver disease (NAFLD) | B6TDN0 | 1 | http://www.kegg.jp/kegg-bin/show_pathway?ko04932+K03946 |
| ko04940 | Type I diabetes mellitus | B4FUE3 | 1 | http://www.kegg.jp/kegg-bin/show_pathway?ko04940+K01580 |
| ko05010 | Alzheimer's disease | B6TDN0 | 1 | http://www.kegg.jp/kegg-bin/show_pathway?ko05010+K03946 |
| ko05012 | Parkinson's disease | B6TDN0 | 1 | http://www.kegg.jp/kegg-bin/show_pathway?ko05012+K03946 |
| ko05016 | Huntington's disease | B6TDN0 | 1 | http://www.kegg.jp/kegg-bin/show_pathway?ko05016+K03946 |
